# The Content of Small 18S rRNA Fragments Is Regulated Developmentally and in Response to Stress in Plants

**DOI:** 10.3390/plants15101512

**Published:** 2026-05-15

**Authors:** Angelina A. Malysheva, Taissiya S. Lopatchenko, Kamilla G. Osikova, Tatyana Kan, Anna S. Nizkorodova, Ruslan V. Kryldakov, Bulat K. Iskakov, Andrey V. Zhigailov

**Affiliations:** 1 M.A. Aitkhozhin Institute of Molecular Biology and Biochemistry, Almaty 050012, Kazakhstan; malysheva.angelina.alx@gmail.com (A.A.M.); t.lopatchenko@su.edu.kz (T.S.L.); kamilla.osikova5@gmail.com (K.G.O.); t.kan@imb-mainz.de (T.K.); anna_niz@mail.ru (A.S.N.); kryldakov@yahoo.com (R.V.K.); bulat.iskakov2@gmail.com (B.K.I.); 2 Institute of Molecular Biology, 55128 Mainz, Germany

**Keywords:** 18S rRNA, discrete fragmentation, SLA-RT-PCR, 40S ribosomal subunit, osmotic stress

## Abstract

Protein synthesis is a crucial biosynthetic process in all organisms, including plants. The integrity of the translational machinery, especially ribosomes, can be compromised during rapid cell division in ontogenesis or in response to environmental stress. In this study, Northern blotting was employed to analyze total RNA from various angiosperms, focusing on small 5′- and 3′-terminal 18S rRNA fragments. Stem-loop array RT-PCR was employed to map the cleavage sites within the target regions. Severe stress, such as extreme drought, induced the accumulation of three distinct 18S rRNA fragments across diverse angiosperm taxa, indicating that this phenomenon is likely universal. In rapidly dividing cells, such as those found in in vitro callus cultures and germinating wheat embryos, high levels of discrete 5′-terminal fragments were observed, while 3′-terminal fragments were absent. The stem-loop array RT-PCR mapping identified specific sites of 18S rRNA strand breaks. Structural annotation of the 3D model of the plant 40S subunit revealed spatial clustering of these sites in proximity to the RPS6 binding region. Notably, wheat cultivars that are tolerant to osmotic stress exhibited significantly higher levels of 18S rRNA fragmentation than sensitive cultivars. This suggests a regulatory mechanism rather than a mere byproduct of apoptotic-like regulated cell death. Additionally, fragmented ribosomes were gradually eliminated during embryo maturation, indicating a process of programmed functional ribophagy. Our findings suggest that a potential inability of plant tissues to selectively retain functional ribosomes might contribute to a decline in generative potential. Monitoring the integrity of the translational machinery could improve breeding efficiency and aid in preserving long-term stored germplasm.

## 1. Introduction

Due to their sessile lifestyle, plants have evolved complex mechanisms to manage stress; however, activating these systems often causes long-term “stress after-effects” that impair fitness after conditions normalize [1]. Under acute lethal stress, plant cells typically undergo necrosis, a process characterized by the uncontrolled collapse of cellular functions. In response to extreme abiotic factors, a series of reactions is triggered in plant tissues that resembles metazoan apoptosis [2]. This process of cellular degradation is commonly referred to as autolytic programmed cell death (AL-PCD) [3]. In cases of non-lethal stress, plant cells remain viable but may experience genomic instability [4,5], epigenetically mediated changes in gene expression [6], and damage to the integrity of cellular components [7]. Even after the stressor is removed, the resulting state can persist for an extended period [8]. Over time, these anomalies can accumulate, leading to cumulative effects. This buildup of detrimental changes may ultimately result in a loss of cellular totipotency.

The accumulation of cellular anomalies is influenced by external factors as well as the dynamics of the cell cycle. During periods of high mitotic activity, the effectiveness of molecular surveillance and error-correction systems is temporarily limited. This limitation allows for rapid cell proliferation to be viewed as a specific form of intracellular stress. Prolonged cultivation leads to a decrease or alteration in the morphogenetic potential of callus tissues [9,10]. In addition to common explanations such as hormonal imbalances or autonomy from external phytohormones [11], other factors contribute to this decline. A significant cause is the replicative machinery’s inability to complete repair processes during rapid cell division. In plant meristems, replicative stress leads to cell-cycle arrest, which is coordinated by the transcription factors E2F and SOG1 during excessive cell division [12,13]. Moreover, the loss of morphogenetic potential in long-term subcultured calli may involve epigenetic “locking,” which occurs via selective DNA hypermethylation or histone modifications at the promoters of embryogenesis-related genes, such as those in the *WUSCHEL* or *BABY BOOM* families [14,15]. Global DNA hypermethylation may further exacerbate this issue [16].

Research indicates that long-term subcultured callus cells exhibit structural changes, including disruptions in the cytoskeleton [17], nucleolus [18], mitochondria [19], and plastids [20]. A significant factor contributing to the declining morphogenetic potential of rapidly dividing cell lines is the disruption of ribosomal structure. Recent findings emphasize the presence of substantial ribosome heterogeneity in eukaryotic cells [21], including those in plants [22]. Unlike animal cells, plant ribosomal protein genes belong to multigene families, with different paralogs expressed in a tissue-specific manner or in response to varying environmental conditions [23]. This heterogeneity is further heightened by intragenomic rDNA polymorphism; long-read sequencing has revealed hundreds of variant rRNA alleles within a single plant, some of which contain substitutions in functionally critical regions [24]. For cells to initiate embryogenesis or organogenesis, they require specific ribosomes capable of translating developmental genes such as *WUSCHEL* or *BBM*. During extended subculturing or chronic stress, changes in the ribosomal pool may prioritize the translation of stress proteins over morphogenetic regulators [25]. Additionally, mutations in ribosomal proteins can lead to meristematic defects because the ribosomes fail to efficiently translate regulatory mRNAs [26]. As a result, the ribosomes in aging cell lines may be unable to translate the specific signals necessary for regeneration.

Furthermore, eukaryotic ribosome heterogeneity extends beyond specific protein alleles and rRNA variants. Post-assembly modifications, such as methylation, oxidation, and limited hydrolysis, can cause structural abnormalities that reduce translational fidelity, leading to misreading and premature termination of translation. The resulting accumulation of aberrant, misfolded proteins induces proteotoxic stress, which may trigger cellular senescence and impede morphogenetic programs. This process mirrors how translation failures contribute to neurodegeneration in aging animals [27] and yeast [28].

Certain toxins further inactivate ribosomes by cleaving rRNA within the subunits. Examples include plant proteins such as ricin, bacterial Shiga toxins, and fungal α-sarcin [29,30]. Specifically, the PiORF4 toxin from *Pichia inositovora* cleaves 28S and 18S rRNA [31]. Based on these observations, we propose a universal mechanism for regulating eukaryotic ribosome efficiency and selectivity under stress through the site-specific cleavage of 18S rRNA within the small (40S) ribosomal subunits.

In previous studies, we demonstrated that stress conditions such as heat shock or amino acid starvation induce specific and non-random cleavage of 18S rRNA in plant cells, resulting in distinct 5′ and 3′ terminal fragments [32,33]. Similar stress-induced 18S rRNA fragments have also been identified in rodent cells and human placental tissue. Whole-transcriptome sequencing further supports the presence of site-specific ribosomal RNA breaks across eukaryotes [34,35,36]. The reproducibility and regulated accumulation of these fragments suggest that 18S rRNA cleavage is a coordinated response rather than merely non-specific degradation.

In this study, we explored 18S rRNA fragmentation throughout plant development and in callus cultures. To verify the precise fragmentation pattern of 18S rRNA, we employed stem-loop array RT-PCR (SLA-RT-PCR). This method utilizes a series of stem-loop reverse transcription primers designed to target potential cleavage sites, ensuring high specificity for fragmented 3′ termini [37]. Our findings emphasize the universality of this discrete fragmentation phenomenon and its potential role in regulating ribosome heterogeneity. We propose that the efficient turnover and recovery of damaged ribosomes are essential for maintaining cellular totipotency and organogenetic capacity. This hypothesis is supported by the observed dynamics of 18S rRNA fragment levels in wheat embryos during caryopsis maturation.

## 2. Materials and Methods

### 2.1. Plasmid Construction and Probe Preparation

Primers for DNA construct assembly and subsequent digoxigenin (DIG)-labeled probe production were designed based on the *Triticum aestivum* L. 18S rRNA sequence (GenBank accession no. AY049040) retrieved from the NCBI GenBank database [38]. De novo synthesized oligonucleotides Ta18S_1-35_Kpn-F (5′-GTACCACAAGCATATGACTACTGGCAGGATCAACCAGGTA)/Ta18S_1-35-Hind-R (5′-AGCTTACCTGGTTGATCCTGCCAGTAGTCATATGCTTGTG) and Ta18S_1776-1810_Kpn-F (5′-GTACCcAATGATCCTTCCGCAGGTTCACCTACGGAAACCTA)/Ta18S_1776-1810-Hind-R (5′-AGCTTAGGTTTCCGTAGGTGAACCTGCGGAAGGATCATTGG) were annealed, phosphorylated, and ligated into the pBluescript KS(+) vector using *Acc65*I and *Hind*III restriction sites. The inserts of DNA constructs Ta5′18S-35nt_pBKS and Ta3′18S-35nt_pBKS were verified via Sanger sequencing using a BigDye™ Terminator v3.1 Cycle Sequencing Kit (Applied Biosystems, Foster City, CA, USA) with the T7 primer (5′-TAATACGACTCACTATAGGG) according to the manufacturer’s protocol. Reaction mixtures were purified using a BigDye XTerminator™ Purification Kit (Applied Biosystems, Foster City, CA, USA) and analyzed on a SeqStudio genetic analyzer (Applied Biosystems, Singapore). The Ta5′18S-35nt_pBKS and Ta3′18S-35nt_pBKS plasmids were linearized with *Hind*III (Thermo Fisher Scientific, Waltham, MA, USA) and transcribed using a DIG RNA Labeling Kit (SP6/T7) (Roche Diagnostics GmbH, Mannheim, Germany) according to the manufacturer’s instructions.

### 2.2. Plant Material and RNA Isolation

Grains at various stages of embryo maturation were removed from the spikes and surface-sterilized in 70% (*v*/*v*) ethanol for 2 min and then in 2% (*w*/*v*) NaOCl for 20 min, and washed thoroughly with sterile water prior to embryo extraction.

To investigate whether stress-induced fragmentation is a universal phenomenon in plants, we examined representatives from various angiosperm families. These included radish (*Raphanus sativus*, Brassicaceae), pea (*Pisum sativum*, Fabaceae), beet (*Beta vulgaris*, Amaranthaceae), tomato (*Solanum lycopersicum*, Solanaceae), pumpkin (*Cucurbita pepo*, Cucurbitaceae), and dill (*Anethum graveolens*, Apiaceae). Each species was subjected to lethal drought stress by incubating 3 g of 10-day-old seedlings on paper filters for 8 h.

To quantify the physiological severity of the desiccation stress, leaf relative water content (*RWC*) was determined according to the method of Barrs and Weatherley [39]. Immediately after the 8 h incubation on filter paper (25 °C; humidity 65%), the detached leaves were weighed to obtain the desiccated weight (*DW*). Subsequently, the samples were immersed in distilled water for 24 h at 4 °C in darkness to reach full turgidity and then weighed to determine the turgid weight (*TW*). Finally, the samples were oven-dried at 80 °C for 48 h to obtain the dry weight (*DRW*). The *RWC* was calculated using the following equation:

(1) $$RWC\left(\%\right)=\frac{\left(FW-DRW\right)\times 100}{TW-DRW},$$

where *FW* is the fresh weight, *TW* is the turgid weight, and *DRW* is the final dry weight.

The water loss rate (*WLR*) was estimated to characterize the kinetics of leaf dehydration. The fresh weight (*FW*) of the detached leaves was measured immediately after excision and after 8 h of incubation on filter paper (*DW*). *WLR* was calculated according to the following formula:

(2) $$WLR\left(mg\;g^{-1}h^{-1}\right)=\frac{\left(FW0h\;-\;FW8h\right)\times 1000}{FW0h\times t},$$

where *FW*_0h_ is the initial fresh weight, *FW*_8h_ is the fresh weight after stress, and *t* is the incubation time in hours.

Total RNA was extracted from 0.1 g of plant tissues (embryos, leaves, seedlings, and calli) using TRIzol reagent (Sigma-Aldrich, St. Louis, MO, USA), following the manufacturer’s instructions. Prior to reverse transcription, RNA isolated with TRIzol was treated with dsDNase (Thermo Fisher Scientific, Waltham, MA, USA), followed by enzyme inactivation at 55 °C for 10 min.

### 2.3. Osmotic Stress Imitation

Three bread wheat (*Triticum aestivum* L.) cultivars with varying levels of stress tolerance were used in this study: ‘Kazakhstanskaya Rannespelaya’ (stress-susceptible), ‘Kazakhstanskaya 10′ (exhibiting increased salinity tolerance), and ‘Kazakhstanskaya 19′ (drought-tolerant). The wheat seeds were surface-sterilized using a 1% sodium hypochlorite solution for 10 min and then thoroughly rinsed with deionized water. The seeds were wrapped in moist gauze (cheesecloth) and germinated in the dark at 25 °C for 48 h. Once uniform germination was achieved, the seedlings were transferred to a hydroponic system with a modified Hoagland’s nutrient solution [40]. The plants were grown in a climate chamber under a 16/8 h light/dark cycle and maintained at a relative humidity of 60%.

On the 9th day of growth, the seedlings from each cultivar were divided into three experimental groups. The control group continued to grow in the basal nutrient solution. To induce osmotic stress, the second group was transferred (shock treatment) to a nutrient solution supplemented with 500 mM sucrose. For salt stress induction, the third group was exposed to 300 mM NaCl added to the nutrient solution (shock treatment). Both stress treatments were maintained under the same environmental conditions as the control group. Samples (shoots) were collected after 3 days of treatment for RNA isolation.

### 2.4. Stem-Loop Array RT-PCR (SLA-RT-PCR)

The SLA-RT-PCR technique was employed to specifically detect cleavage sites during the fragmentation of 18S rRNA. In this method, an array of stem-loop primers was used for reverse transcription, followed by PCR amplification to map the 3′-termini of the resulting fragments [37]. Each 20 μL reaction mixture for cDNA synthesis consisted of 1 μg of total RNA, 20 pmol of the corresponding reverse stem-loop primers (Supplementary Material S1), 4 μL of 5X RT Buffer, 0.5 mM of each dNTP, 20 U of Thermo Scientific™ RiboLock RNase Inhibitor (Thermo Fisher Scientific, Waltham, MA, USA), and 200 U of Maxima Reverse Transcriptase (Thermo Fisher Scientific, Waltham, MA, USA). The temperature conditions for this process were as follows: 25 °C for 5 min, 50 °C for 30 min, and 85 °C for 5 min. For the positive controls, reverse primers Ctrl-166-R (targeting 5′-terminal 18S rRNA fragments) and Ctrl-1749-R (targeting 3′-terminal 18S rRNA fragments) were used (Supplementary Material S1).

Amplification was carried out using Hot Start Taq DNA Polymerase (New England BioLabs, Ipswich, MA, USA) in accordance with the manufacturer’s instructions. Primer 5(75)-1-F served as the forward primer for the 5′-terminal 18S rRNA fragments, while primer 3(110)-1434-F was used for the 3′ -terminal fragments (Supplementary Material S1). The SLA-PCR-UniRev primer, which is complementary to the hairpin structure of the primers used for reverse transcription, was employed as the reverse primer in both instances (Supplementary Material S1). In the case of positive controls, the same reverse primers used in the reverse transcription step were utilized. Each 20 μL reaction contained 2.0 μL of the reverse transcription reaction product. The thermal cycling conditions included a 95 °C pre-denaturation step for 5 min, followed by 40 PCR cycles consisting of 94 °C for 25 s, 56 °C for 25 s, and 72 °C for 1 min, concluding with a final incubation at 72 °C for 5 min. The PCR products were analyzed using 1.5% agarose gel electrophoresis and visualized under UV light.

To generate the *Ta18S-3′end-1698/1699* control RNA for method validation, a 282 bp PCR product was amplified from total wheat RNA using reverse transcription polymerase chain reaction (RT-PCR) with the primers 3(110)-1434-Acc-F and 3(110)-1698-ER-R (Supplementary Material S1). The resulting fragment was then digested with the restriction endonucleases *Acc65*I and *EcoR*I and cloned into the corresponding sites of the pBluescript KSII (+) vector. The resulting construct, *Ta18S-3′end-1698/1699-pBKS*, was linearized with *EcoR*I and subsequently used as a template for in vitro transcription of the *Ta18S-3′end-1698/1699* control RNA. This transcription was carried out using T7 RNA polymerase (Thermo Fisher Scientific, Waltham, MA, USA) according to the manufacturer’s protocol. Finally, the synthesized RNA was treated with DNase I (New England BioLabs, Ipswich, MA, USA) according to the manufacturer’s protocol and precipitated using 3 M LiCl (Sigma-Aldrich, St. Louis, MO, USA).

### 2.5. Northern Blotting

RNA samples (5 μg) were denatured in a formamide solution containing 0.05% bromophenol blue by heating at 80 °C for 2 min. They were then chilled on ice and loaded onto an 8% polyacrylamide gel containing 8 M urea. Electrophoresis was performed in 1× TAE buffer at a constant voltage of 150 V, initially at a rate of 8 V/cm for 15 min, followed by 6 V/cm for 3 to 4 h. RNAs were transferred to a Hybond N+ nylon membrane (GE Healthcare Amersham, Little Chalfont, UK) equilibrated in 0.1× TAE using a semi-dry blotter (C.B.S. Scientific, San Diego, CA, USA) at 250 mA for 30 min. The membrane was rinsed with 70% ethanol, dried, and irradiated with 1200 mJ/cm^2^ of 254 nm shortwave UV light in a crosslinker (UVP, Upland, CA, USA).

Prehybridization was carried out at 58 °C for 1 h in a hybridization solution (Roche Diagnostics GmbH, Mannheim, Germany). Hybridization was subsequently performed overnight at 58 °C using 50 ng/mL of a DIG-labeled RNA probe. To remove nonspecific binding, membranes were washed twice in 2 × SSC/0.1% SDS (15 min per wash) and twice in 0.1 × SSC/0.1% SDS (15 min per wash) at 58 °C. For immunological detection, membranes were blocked for 1 h in 2% blocking buffer and incubated with anti-digoxigenin-AP Fab fragments (Roche Diagnostics GmbH, Mannheim, Germany) at a 1:5000 dilution in blocking buffer. After equilibration in detection buffer, chemiluminescent signals were generated using the CDP-Star substrate (Merck/Novagen, Gillingham, UK) and visualized on X-ray film (Agfa HealthCare NV, Mortsel, Belgium).

### 2.6. Bioinformatics, Densitometry, and Statistical Analyses

To semi-quantitatively assess the degree of discrete 18S rRNA fragmentation, blots were analyzed via densitometry using ImageJ software version 1.42q (National Institutes of Health, Bethesda, MD, USA). All measurements were performed in at least three independent replicates. The densitometric signal intensities of the target bands were normalized to the intensity of the 5S rRNA internal loading control. Data are presented as the mean ± standard error of the mean (SEM). Statistical significance was evaluated using Student’s *t*-test, with differences considered significant at *p* < 0.05.

The structure of the actively translating *Nicotiana tabacum* 80S ribosome, which has a 2.2 Å resolution (PDB ID: 8AUV [41]), was utilized in Swiss-PDB-Viewer to map rRNA disruption sites within the composition of the plant’s 40S ribosomal subunits. Data on the secondary structure of 18S rRNA were obtained from the Comparative RNA Web (CRW) site [42].

## 3. Results

We previously identified two 5′-coterminal fragments of 18S rRNA, namely, 5′18S132rRF (130–135 nt) and 5′18S75rRF (75–80 nt), along with one 3′-terminal fragment, 3′18S100rRF (100–105 nt). These fragments were almost undetectable in young, non-stressed wheat plants; however, their levels significantly increased under conditions of amino acid starvation and heat shock [32,33].

### 3.1. Testing the Universality of the Phenomenon in Angiosperms

To assess whether the aforementioned 5′- and 3′-fragments of 18S rRNA are also present in dicots and to explore the universality of this occurrence, we analyzed the effect of lethal drought stress on 18S rRNA integrity in 10-day-old seedlings from six different dicot families using Northern blotting. The control plants were not exposed to any stress. Table 1 presents the water relation parameters of plant material under osmotic stress. More detailed information regarding the parameters assessing water loss in plants due to osmotic stress can be found in Supplementary Material S2.

**Figure 1:**
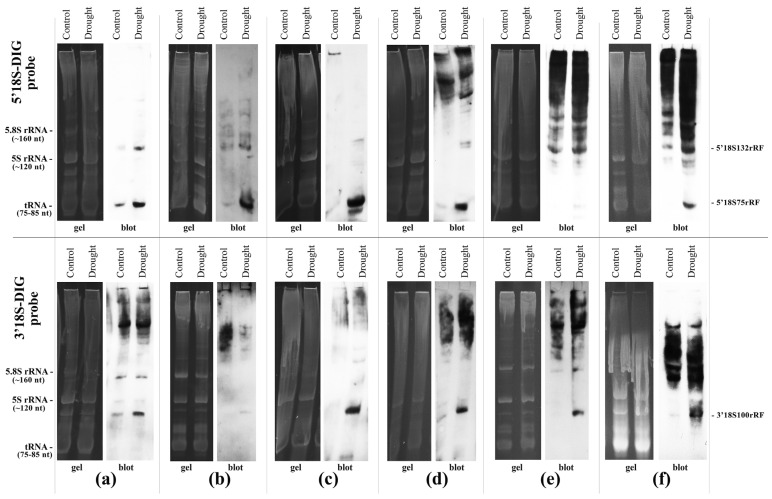
Northern blot analysis of 18S rRNA 5′- and 3′-terminal fragment profiles in representatives of various angiosperm families under control conditions and lethal drought stress: (**a**) radish (*Raphanus sativus*); (**b**) pea (*Pisum sativum*); (**c**) beet (*Beta vulgaris*); (**d**) tomato (*Solanum lycopersicum*); (**e**) pumpkin (*Cucurbita pepo*); (**f**) dill (*Anethum graveolens*). The upper and lower panels show hybridization data using “5′18S-DIG” and “3′18S-DIG” probes, respectively.

The results of Northern blotting analysis are presented in Figure 1.

As illustrated in Figure 1, specific discrete fragments of the 18S rRNA, namely, 5′18S132rRF, 5′18S75rRF, and 3′18S100rRF, were detected in all the plant species tested after exposure to lethal drought stress. These findings suggest that the fragmentation of 18S rRNA is a universal phenomenon, at least among angiosperms.

### 3.2. Mapping of 18S rRNA Cleavage Sites Using the SLA-RT-PCR Approach

**Figure 2:**
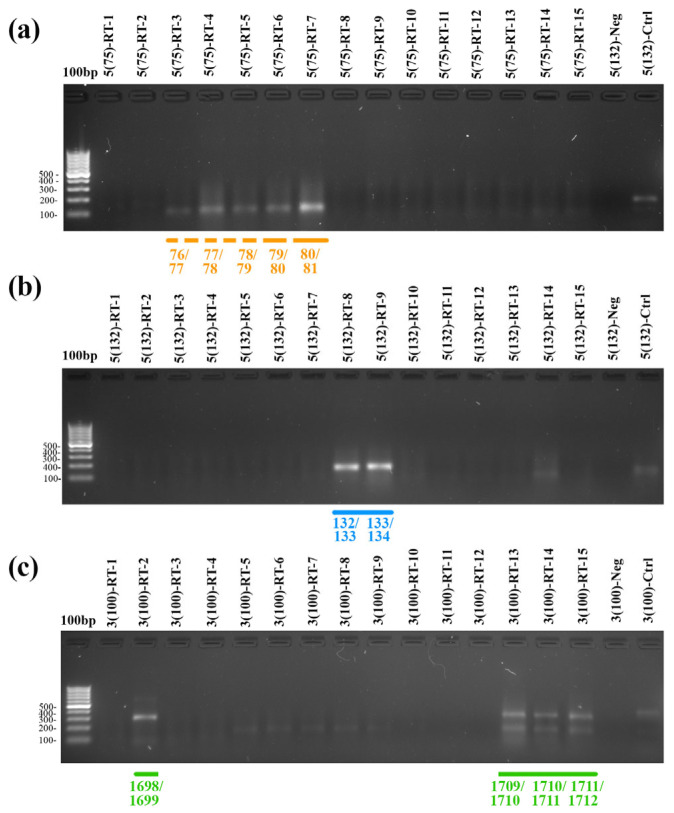
Electrophoretic analysis of SLA-RT-PCR amplification products. The 1.5% agarose gels are shown (each panel represents a specific target region of the 18S rRNA). Corresponding positions in the 18S rRNA molecule where cleavage occurs are indicated at the bottom of each panel. (**a**) Testing of the 75–89 region (using primers 5(75)-1-F/SLA-PCR-UniRev for PCR); (**b**) testing of the 126–140 region (using primers 5(75)-1-F/SLA-PCR-UniRev for PCR); (**c**) testing of the 1698–1712 region (using primers 3(110)-1434-F/SLA-PCR-UniRev for PCR). The names of the primers used for reverse transcription are labeled above the lanes. The 100 bp DNA ladder is included for reference. ‘5(135)-Ctrl’: positive control for 5′-coterminal 18S rRNA fragments, which uses wheat RNA with the primer Ctrl-166-R for reverse transcription, and primers 5(75)-1-F and Ctrl-166-R for PCR. ‘5(135)-Neg’: negative control for 5′-coterminal 18S rRNA fragments, using no RNA, the primer Ctrl-166-R for reverse transcription, and primers 5(75)-1-F and Ctrl-166-R for PCR. ‘3(100)-Ctrl’: positive control for 3′-terminal 18S rRNA fragments, utilizing wheat RNA, the primer Ctrl-1749-R for reverse transcription, and primers 3(110)-1434-F and Ctrl-1749-R for PCR. ‘3(100)-Neg’: negative control for 3′-terminal 18S rRNA fragments, also using no RNA, the primer Ctrl-1749-R for reverse transcription, and primers 3(110)-1434-F and Ctrl-1749-R for PCR. The primer sequences can be found in Supplementary Material S1.

The SLA-RT-PCR method has been successfully employed for the precise mapping of microRNA precursor cleavage sites [37]. We applied this technique to identify the cleavage sites of 18S rRNA under stress conditions. To map the cleavage in each target region, we synthesized 15 hairpin-forming reverse primers, each containing six single-stranded nucleotides at the 3′-end. The first study region encompassed nucleotide residues 75–89, the second covered residues 126–140, and the third included residues 1698–1712 (nucleotide positions for *T. aestivum* L. 18S rRNA correspond to GenBank reference sequence AY049040). The total RNA used as the template was isolated from 10-day-old wheat seedlings subjected to lethal stress (dehydration resulting in a lack of recovery after rewatering), which involved dehydration followed by a lack of recovery after rewatering. The results of the analysis are presented in Figure 2. Validation of the SLA-RT-PCR method for mapping disruption sites in plant 18S rRNA molecules, including the negative control reaction, is provided in Supplementary Material S3.

**Figure 3:**
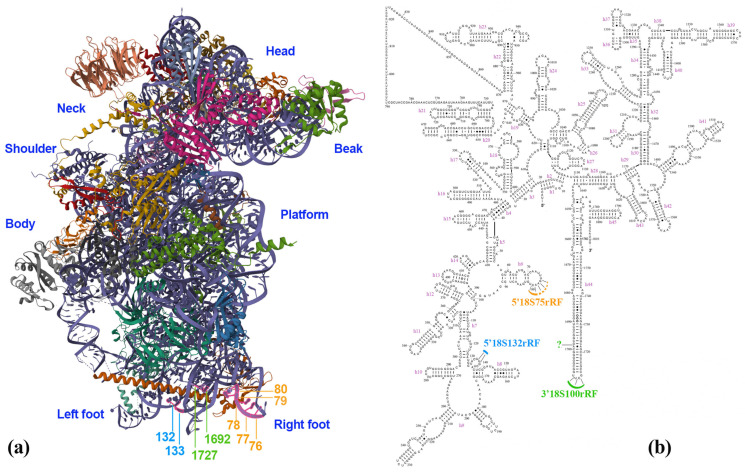
Positioning of 18S rRNA breakpoints at key sites. (**a**) Cryo-electron microscopy (Cryo-EM) structure of the plant (*Nicotiana tabacum*) 40S subunit (PDB ID: 8AUV [41]). Nucleotide residues that interact with RPS6 are highlighted. (**b**) The secondary structure of the plant *Triticum aestivum* L. 18S rRNA, based on model XR_006476226 [42]. Key cleavage sites of the 18S rRNA identified via SLA-RT-PCR are indicated: the 75–89 region is highlighted in orange, the 126–140 region in blue, and the 1698–1712 region in green. The question mark ("?") indicates the cleavage gap between residues 1698 and 1699; notably, the resulting cleavage product was not detectable by northern blot analysis. Note that the segment 1696–1726, which includes the target 1698–1712 region, was absent from the 3D atomic model of the 40S subunit. Consequently, the flanking residues 1692 and 1727 are marked in green instead.

Figure 3 shows the secondary and tertiary (3D) structures of plant 18S rRNA within the 40S ribosomal subunit, with the cleavage sites annotated based on SLA-RT-PCR analysis.

We found that under severe lethal stress, cleavages occur within the 75–89 nucleotide region of wheat 18S rRNA. These cleavages occur between residues 76/77, 77/78, 78/79, 79/80, and 80/81 (helix h6, Figure 3b), with the cleavage between residues 80/81 being the most predominant (Figure 2a). In the 126–140 nucleotide region, we identified two cleavage sites with similar efficiency between residues 132/133 and 133/134, which are located in the eukaryotic expansion segment between helices h7 and h8 (Figure 3b). Within the 3′-terminal region (nucleotides 1698–1712), we detected three cleavage sites in the loop of the h44 hairpin (Figure 3b) at positions 1709/1710, 1710/1711, and 1711/1712 (Figure 2c). Additionally, another cleavage was observed between residues 1698 and 1699 (Figure 2c). The 10-nucleotide distance between this site and the h44 loop suggests that differentiation of this fragment from 3′18S100rRF should theoretically be possible. However, such longer (~113 nt) 3′-terminal fragments have not been previously observed in blots. It is likely that cleavage at this position occurs during later stages, after most 18S rRNA molecules have already been processed at the h44 loop. In this case, the cleavage results in a short 10-nucleotide internal fragment that lacks the 3′-terminus, making it undetectable by probes specific to the 18S rRNA 3′-terminal sequence.

As a result, no single precise cleavage position was identified in any of the target regions of the 18S rRNA molecule. These findings indicate that the fragmentation of 18S rRNA induced by stress is less influenced by the primary RNA sequence, rather more by its specific spatial location and/or conformation within the 40S ribosomal subunit.

### 3.3. A Stress-Induced Response or a Consequence of Cellular Autolysis?

A fundamental question arises: is the discrete fragmentation of 18S rRNA observed under stress a regulatory mechanism designed to downregulate protein synthesis promptly, or is it a symptomatic consequence of cell death? To address this question, three contrasting cultivars of spring bread wheat were subjected to two types of non-lethal osmotic stress (allowing for post-stress recovery): 0.5 M sucrose and 0.3 M NaCl treatments. The latter is considered more severe, as the osmotic component is compounded by the cytotoxic impact of sodium ions on cellular membranes [43]. The experiment utilized the following cultivars: ‘Kazakhstanskaya Rannespelaya’ (early-maturing and stress-sensitive), ‘Kazakhstanskaya 10’ (characterized by moderate drought and heat tolerance, as well as high salt tolerance), and ‘Kazakhstanskaya 19’ (noted for high drought tolerance and moderate salt tolerance) [44].

**Figure 4:**
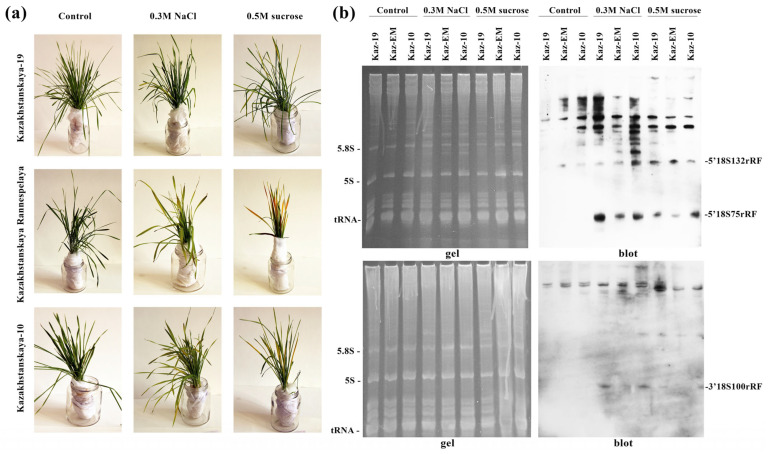
Assessment of the accumulation of target stress-induced 18S rRNA fragments under osmotic stress in contrasting wheat (*Triticum aestivum* L.) cultivars. (**a**) Phenotypes of control wheat seedlings (‘Control’) of three varieties and those exposed to 0.3 M sodium chloride (‘0.3 M NaCl’) and 0.5 M sucrose (‘0.5 M sucrose’) for three days. (**b**) Northern blot analysis. The upper panel shows an 8% polyacrylamide gel containing 8 M urea and 1x TAE (left) and the corresponding blot (right) hybridized with the 5′18S-DIG probe. The lower panel displays the gel and blot using the 3′18S-DIG probe. Kaz-19, ‘Kazakhstanskaya 19’; Kaz-10, ‘Kazakhstanskaya 10’; Kaz-EM (Early Maturing), ‘Kazakhstanskaya Rannespelaya’; 5.8S, 5.8S rRNA; 5S, 5S rRNA.

The experimental results are presented in Figure 4. As anticipated, seedlings of the stress-sensitive cultivar ‘Kazakhstanskaya Rannespelaya’ exhibited pronounced morphological symptoms under both high salinity and sucrose-induced osmotic stress (Figure 4). In contrast, both tolerant cultivars exhibited only minimal phenotypic signs of distress.

To determine whether the severity of stress symptoms correlates with the level of discrete rRNA fragmentation, Northern blot analysis of the total RNA was performed (Figure 4b). The densitometric analysis data are summarized in Table 2. Under control conditions, the levels of 18S rRNA fragments (5′18S132rRF, 5′18S75rRF, and 3′18S100rRF) were negligible across all cultivars but increased significantly under stress. Notably, under both types of osmotic stress, the accumulation of 5′18S75rRF and 3′18S100rRF fragments in the tolerant cultivars (‘Kazakhstanskaya 19’ and ‘Kazakhstanskaya 10’) was severalfold higher than in the sensitive ‘Kazakhstanskaya Rannespelaya’ (Table 2). More detailed data on the densitometric analysis are provided in the Supplementary Materials S2.

Consequently, an inverse correlation was observed between the severity of phenotypic damage (chlorosis, leaf wilting, and growth retardation) and the level of discrete 18S rRNA fragmentation.

### 3.4. Discrete Fragmentation of 18S rRNA During Rapid Cell Proliferation

**Figure 5:**
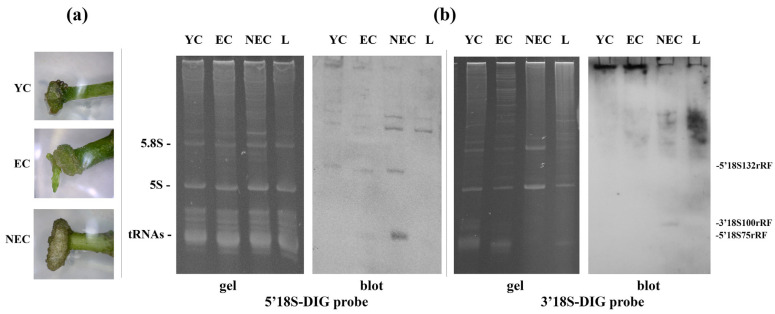
Analysis of small 5′- and 3′-terminal 18S rRNA fragment profiles in rapidly dividing in vitro cells of potato (*Solanum tuberosum*). (**a**) Callus morphology; (**b**) Northern blot analysis. YC, young callus; EC, mature embryogenic callus; NEC, non-embryogenic callus; L, young leaf mesophyll; 5.8S, 5.8S rRNA; 5S, 5S rRNA.

Plant cell division rates vary significantly. While extremely rapid proliferation occurs in germinating embryos, near-maximal rates can be achieved in in vitro callus cultures using phytohormones and nutrient-rich media. Given that rapid cell division itself may act as a form of physiological stress, we investigated whether the levels of stress-induced small 5′ and 3′-terminal 18S rRNA fragments increase in such cells. Furthermore, we compared the fragmentation profiles between embryogenic and non-embryogenic calli. The experimental results are shown in Figure 5.

As anticipated, none of the targeted small terminal 18S rRNA fragments were detected in young leaf mesophyll cells. However, the 5′18S132rRF fragment was found in young calli, and both 5′-terminal 18S rRNA fragments (5′18S75rRF and 5′18S132rRF) were identified in long-term cultured calli (Figure 5). Densitometric analysis indicated that the relative abundance of the 5′18S75rRF fragment was significantly higher in non-embryogenic calli than in embryogenic calli (*p* = 0.0023). The 3′-terminal fragment, 3′18S100rRF, was detected in measurable quantities only within non-embryogenic callus cells (Figure 5).

**Figure 6:**
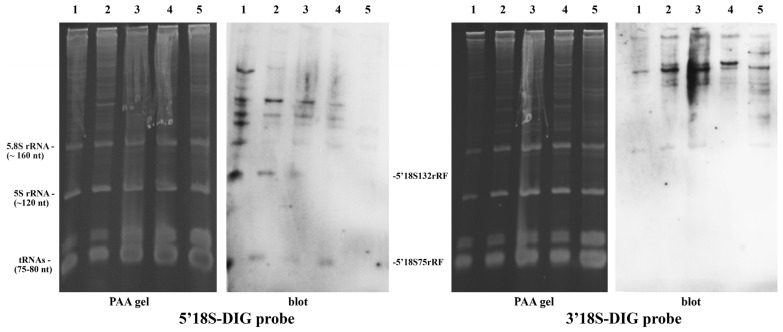
Investigation of the discrete 18S rRNA fragmentation phenomenon in wheat embryos (*Triticum aestivum* L., cv. ‘Kazakhstanskaya 10′) at different stages of caryopsis maturation: (1) germinated embryos; (2) milk ripe stage; (3) soft-dough (milk–wax) stage; (4) hard-dough (wax–ripe) stage; (5) mature caryopsis.

This indicates that specific 18S rRNA fragments accumulate in actively dividing callus cells. However, because cell culture is an artificial environment where cells can be subjected to oxidative and other stresses during cultivation on nutrient media [45], we aimed to investigate the accumulation patterns of these fragments under natural physiological conditions. To achieve this, we monitored levels of 18S rRNA fragments during the maturation of wheat caryopses. Samples were collected at various developmental stages, namely milk ripeness, soft dough (milk–wax), hard dough (wax), and mature caryopsis stages, as well as from fully mature and germinating seeds. The experimental results are presented in Figure 6.

The 3′-terminal fragment 3′18S100rRF was not detected in any of the samples (Figure 6). This observation is consistent with our previous findings, as this specific 18S rRNA fragment appeared only under severe, lethal, and sublethal stress conditions, and remained absent under physiologically normal conditions [33]. Similarly, this fragment was undetectable in young and mature embryogenic calli, appearing only in non-embryogenic calli (Figure 6). Regarding the 5′-terminal 18S rRNA fragments (5′18S75rRF and 5′18S132rRF), their levels were relatively high in embryos of germinating seeds and milk-stage caryopses. However, as the caryopsis matured, the concentration of these fragments in wheat embryos gradually decreased until their complete disappearance in mature grains (Figure 6). This is a crucial finding, as it suggests that a selection process for ribosomal integrity likely occurs during embryo maturation. Only ribosomes with intact rRNA are preserved, while others presumably undergo targeted ribophagy. It appears that the presence of intact, fully functional ribosomes is critically important for the successful development of the embryo into a viable plant.

## 4. Discussion

We observed that lethal stress (severe drought) induces 18S rRNA fragmentation in the cells of various monocotyledonous and dicotyledonous angiosperm species. Notably, the prevalence of fragments of strictly defined sizes indicates a discrete fragmentation pattern of this molecule. This raised the question of whether this universal phenomenon is a direct consequence of AL-PCD or an adaptive mechanism aimed at suppressing mRNA translation in response to stress.

To address this, we evaluated the response of resistant and susceptible wheat cultivars to osmotic stress. Our results demonstrated that salt- and drought-tolerant cultivars, which exhibited no significant morphological signs of damage under simulated osmotic stress (elevated NaCl and sucrose concentrations), accumulated significantly higher levels of target 5′- and 3′-terminal 18S rRNA fragments than the contrasting susceptible cultivar. Conversely, the susceptible wheat plants exhibited marked growth inhibition and necrosis under the same experimental conditions. Notably, despite these symptoms, all tested plants remained viable and exhibited the capacity for recovery upon return to control conditions, indicating that the observed fragmentation occurred within living cells. The inverse correlation between physiological damage and fragment accumulation further supports the conclusion that the generation of specific 18S rRNA fragments most likely serves as a stress adaptation mechanism, potentially acting through global or selective inhibition of mRNA translation.

The observation that osmotic stress triggers the formation of similar 18S rRNA fragments across diverse angiosperm species suggests that this stress-induced process is a universal phenomenon in plants.

Notably, several protein biosynthesis suppression mechanisms that are well established in yeast and mammals appear to be absent in plant cells. Specifically, plants do not exhibit endogenous kinase activity toward eEF2 under either basal [46] or stress conditions [47]. Furthermore, no clear homologs of eIF4E-binding proteins (4E-BPs) have been identified in plants [48], nor have any orthologs of 4E-BP genes been found in plant genomes [49]. Regarding the core translational global repression mechanism in yeast and mammals—the inactivation of the GTP-exchange factor eIF2B via eIF2α phosphorylation [50]—it has been demonstrated that ternary complex recycling in plant cells can occur independently of eIF2B [51]. Additionally, in vitro eIF2α phosphorylation in plant systems does not result in potent inhibition of protein synthesis [52]. Of the four primary eIF2α kinases found in mammals (mPKR, mHRI, mPERK, and mGCN2), only pGCN2 is present in plants, and its phosphorylation of peIF2α is not a universal response to all stress types [53].

Consequently, the translational control mechanisms involving the phosphorylation of initiation and elongation factors, while prominent in mammals and yeast, are either limited or non-functional in plant cells. Nevertheless, the timely suppression of mRNA translation remains a critical requisite for maintaining cell viability under stress. We propose that rapid, stress-induced ribosomal damage—specifically the cleavage of 18S rRNA within the 40S subunit—serves as a universal mechanism in plants to abruptly halt protein synthesis, thereby conserving energy and amino acids while preventing the accumulation of misfolded proteins. Osmotic stress is a significant factor that limits crop productivity, with salinity presenting a serious challenge by causing both immediate water deficiency and subsequent ion toxicity [54]. While osmotic stress, mediated by substances like NaCl, is known to strongly inhibit translation [55,56,57] without leading to eIF2α phosphorylation [52,58,59], our study suggests that the observed site-specific cleavage of 18S rRNA may be a mechanism for this inhibition, even though a direct correlation could not be definitively established.

In vitro plant tissue culture technologies are considered a viable alternative to conventional propagation and cultivation methods for planned long-duration spaceflight. However, the decline in regenerative potential observed in long-term cell cultures poses a significant challenge to such applications. While numerous hypotheses currently exist to explain this phenomenon, the loss of translational machinery integrity—specifically, the cleavage of the 18S rRNA molecule within the 40S ribosomal subunit at key positions—may also be a major contributing factor.

Using wheat as a model, we demonstrated that the proportion of ribosomes containing 18S rRNA breaks at specific positions in regions 75–89 and 126–140 gradually decreases until their complete disappearance during grain maturation. We hypothesize that the capacity of plants to eliminate defective ribosomes promptly is a key determinant of post-stress recovery. Failure to achieve such cellular ‘self-cleansing’ likely results in programmed cell death or the loss of totipotency, ultimately leading to diminished regenerative potential in the affected tissues. Further investigation into targeted ribophagy pathways, such as non-functional rRNA decay (NRD) or ribosome collision-induced degradation [60,61], as well as identifying methods for their activation, may offer strategies to mitigate the loss of regenerative capacity in long-term callus cultures.

It should be noted that the SLA-RT-PCR method cannot inherently distinguish between regulated cleavage and non-specific RNA degradation, as both processes may generate identical 3′-termini. However, several factors suggest that the observed 18S rRNA fragmentation is a specific, regulated process. The consistent detection of discrete, reproducible bands, rather than a continuous smear, across different species, and the clear correlation between fragment abundance and specific stress treatments, indirectly indicate that these disruption sites result from targeted cleavage rather than random RNA decay.

After annotating the identified cleavage sites of 18S rRNA within three key regions of the 3D structure of the plant 40S ribosomal subunit, we found that these sites are clustered near the binding site of the ribosomal protein RPS6 (Figure 3a) [42]. This clustering may be coincidental, or it could suggest that RPS6 plays a role in stress-induced 18S rRNA cleavage, either directly or indirectly through trans-acting factors. Additionally, the phosphorylation status of RPS6 might influence this process. The potential involvement of this important ribosomal protein in stress-induced fragmentation of 18S rRNA in plants deserves further investigation.

This study has several limitations. Specifically, a direct correlation was not established between cleavage of the 18S rRNA molecule within the 40S ribosomal subunits and the specific level of total or selective inhibition of mRNA translation.

## 5. Conclusions

This study examined the process of discrete fragmentation of 18S rRNA in plants. We demonstrated that rapid mitotic division and abiotic stress—both lethal and sublethal osmotic stress—induce site-specific cleavage of the 18S rRNA within the 40S ribosomal subunit at three targeted regions. Notably, stress-tolerant genotypes exhibited higher levels of 18S rRNA fragmentation compared to sensitive ones. Using the SLA-RT-PCR approach, we mapped these cleavage sites, and structural annotation of the 3D model of the plant 40S subunit revealed that these sites cluster in proximity to the RPS6 binding region.

We propose that discrete fragmentation of 18S rRNA may serve as a universal mechanism for translational inhibition or selective mRNA translation in plants. The ability of plant cells to rapidly eliminate or turn over damaged components of the translational machinery may be a key factor in determining their regenerative potential. Furthermore, the role of discrete 18S rRNA fragmentation as an adaptive stress response likely extends beyond the plant kingdom, warranting further comprehensive investigation.

## Figures and Tables

**Table 1:** Relative water content (*RWC*) and water loss rate (*WRL*) of excised leaves from six cultivated plant species after 8 h of dehydration under controlled conditions (25 °C, 60% relative humidity).

Common Name	Scientific Name	Initial Relative Water Content (%) *	Relative Water Content After 8 h Dehydration (%) *	Water Loss Rate (mg·g^−1^·h^−1^) *
Radish	*Raphanus sativus*	88.88 ± 1.07	55.91 ± 0.91	54.72 ± 0.61
Pea	*Pisum sativum*	88.1 ± 0.39	70.03 ± 0.27	35.56 ± 0.73
Beet	*Beta vulgaris*	87.43 ± 0.46	72.9 ± 0.3	23.33 ± 0.64
Tomato	*Solanum lycopersicum*	85.81 ± 0.21	62.26 ± 0.35	36.94 ± 0.97
Pumpkin	*Cucurbita pepo*	90.87 ± 0.39	52.24 ± 0.48	56.53 ± 0.37
Dill	*Anethum graveolens*	85.36 ± 0.6	54.16 ± 0.59	53.75 ± 0.64

* Data represent the mean ± standard error (SEM) of three independent replicates.

**Table 2:** Densitometric analysis of 18S rRNA discrete fragmentation levels in wheat cultivars under osmotic stress.

Stress	‘Kazakhstanskaya Rannespelaya’ ^1^	‘Kazakhstanskaya 19’		‘Kazakhstanskaya 10’	
		Relative Density	*p*	Relative Density	*p*
S_5′18S75rRF_/S_5S RNA_					
Control ^1^	0.084 ± 0.063	0.129 ± 0.08	0.27718	0.084 ± 0.042	0.97853
300 mM NaCl	4.037 ± 0.271	8.505 ± 0.565	0.0075 *	4.416 ± 0.233	0.2435
*p*	0.0032 *	0.0034 *		0.002 *	
500 mM Sucrose	0.373 ± 0.033	2.667 ± 0.227	0.0071 *	3.085 ± 0.147	0.0018 *
*p*	0.0109 *	0.0036 *		0.0019 *	
					
S_5′18S132rRF_/S_5S RNA_					
Control ^1^	0.589 ± 0.232	0.195 ± 0.034	0.2323	0.337 ± 0.084	0.2467
300 mM NaCl	1.135 ± 0.124	0.585 ± 0.079	0.0134 *	3.201 ± 0.296	0.0123 *
*p*	0.0771	0.0352 *		0.0061 *	
500 mM Sucrose	1.486 ± 0.081	3.392 ± 0.381	0.0239 *	0.766 ± 0.042	0.0046 *
*p*	0.0342 *	0.0123 *		0.0189 *	
					
(S_5′18S132rRF_/S_5S RNA_) + (S_5′18S75rRF_/S_5S RNA_)					
Control ^1^	0.675 ± 0.287	0.324 ± 0.096	0.2305	0.421 ± 0.123	0.2626
300 mM NaCl	5.173 ± 0.386	9.09 ± 0.641	0.009 *	7.618 ± 0.521	0.0321 *
*p*	0.0047 *	0.0039*		0.0031 *	
500 mM Sucrose	1.859 ± 0.114	6.059 ± 0.606	0.0135 *	3.851 ± 0.188	0.002 *
*p*	0.0254 *	0.0078*		0.0014 *	
					
S_3′18S100rRF_/S_5S RNA_					
Control ^1^	0.079 ± 0.006	0.059 ± 0.006	0.1317	0.063 ± 0.0217	0.6087
300 mM NaCl	0.363 ± 0.055	1.395 ± 0.059	0.0021 *	1.758 ± 0.078	0.0076 *
*p*	0.0327 *	0.0018 *			
500 mM Sucrose	0.148 ± 0.014	0.388 ± 0.0419	0.0154 *	0.456 ± 0.058	0.0245 *
*p*	0.0335 *	0.0188 *			

^1^ Reference parameter. * Significant difference (*p* < 0.05). Data are presented as the mean ± standard error of the mean (SEM).

## Data Availability

The original contributions presented in the study are included in the article/supplementary material, further inquiries can be directed to the corresponding author.

## Supplementary Materials

The following supporting information can be downloaded at: https://www.mdpi.com/article/10.3390/plants15101512/s1. Supplementary Material S1: Primers used in the SLA-RT-PCR; Supplementary Material S2: Statistical Analysis; Supplementary Material S3: Validation of SLA-RT-PCR method for mapping disruption sites of plant 18S rRNA molecules.

## Abbreviations

The following abbreviations are used in this manuscript:

18S rRNA	18S ribosomal RNA
AL-PCD	Autolytic Programmed Cell Death
4E-BPs	The eIF4E-binding proteins
Cryo-EM	Cryo-electron microscopy
DIG	Digoxigenin
NRD	Non-functional rRNA Decay
SLA-RT-PCR	Stem-loop array reverse transcription polymerase chain reaction

## References

[1] Crisp P.A., Ganguly D., Eichten S.R., Borevitz J.O., Pogson B.J. (2016). Reconsidering plant memory: Intersections between stress recovery, RNA turnover, and epigenetics. Sci. Adv..

[2] Galluzzi L., Bravo-San Pedro J.M., Vitale I., Aaronson S.A., Abrams J.M., Adam D., Alnemri E.S., Altucci L., Andrews D., Annicchiarico-Petruzzelli M. (2015). Essential versus accessory aspects of cell death: Recommendations of the NCCD 2015. Cell Death Differ..

[3] Reape T.J., McCabe P.F. (2008). Apoptotic-like programmed cell death in plants. New Phytol..

[4] Szurman-Zubrzycka M., Jędrzejek P., Szarejko I. (2023). How do plants cope with DNA damage? A concise review on the DDR pathway in plants. Int. J. Mol. Sci..

[5] Manova V., Gruszka D. (2015). DNA damage and repair in plants—from models to crops. Front. Plant Sci..

[6] Kinoshita T., Seki M. (2014). Epigenetic memory for stress response and adaptation in plants. Plant Cell Physiol..

[7] Mittler R. (2017). ROS are good. Trends Plant Sci..

[8] Zhu J.K. (2016). Abiotic stress signaling and responses in plants. Cell.

[9] Gaspar T., Kevers C., Bisbis B., Franck T., Crevecoeur M., Greppin H., Dommes J. (2000). Loss of plant organogenic totipotency in the course of *in vitro* neoplastic progression. Vitr. Cell Dev. Biol. Plant..

[10] Bohorova N.E., Pfeiffer W.H., Mergoum M., Crossa J., Pacheco M., Estañol P. (2001). Regeneration potential of CIMMYT durum wheat and triticale varieties from immature embryos. Plant Breed..

[11] Christou P. (1988). Habituation in *in vitro* soybean cultures. Plant Physiol..

[12] Nisa M., Eekhout T., Bergis C., Pedroza-Garcia J.A., He X., Mazubert C., Vercauteren I., Cools T., Brik-Chaouche R., Drouin-Wahbi J. (2023). Distinctive and complementary roles of E2F transcription factors during plant replication stress responses. Mol. Plant.

[13] Serrano-Mislata A., Hernández-García J., de Ollas C., Blanco-Touriñán N., Jurado-García S., Úrbez C., Gómez-Cadenas A., Sablowski R., Alabadí D., Blázquez M.A. (2025). Growth arrest is a DNA damage protection strategy in *Arabidopsis*. Nat. Commun..

[14] Sun B., Zhou Y., Cai J., Shang E., Yamaguchi N., Xiao J., Looi L.S., Wee W.Y., Gao X., Wagner D. (2019). Integration of transcriptional repression and polycomb-mediated silencing of WUSCHEL in floral meristems. Plant Cell.

[15] Hesami M., Pepe M., Spitzer-Rimon B., Eskandari M., Jones A.M.P. (2025). Epigenetic factors related to recalcitrance in plant biotechnology. Genome.

[16] Tomasiak A., Sala-Cholewa K., Berg L.S., Braszewska A., Betekhtin A. (2023). Global epigenetic analysis revealed dynamic fluctuations in levels of DNA methylation and histone modifications in the calli of *Fagopyrum* with different capacity for morphogenesis. Plant Cell Tiss. Organ. Cult..

[17] Wasteneys G.O., Yang Z. (2004). New views on the plant cytoskeleton. Plant Physiol..

[18] Kalinina N.O., Makarova S., Makhotenko A., Love A.J., Taliansky M. (2018). The multiple functions of the nucleolus in plant development, disease and stress responses. Front Plant Sci..

[19] Fukui H., Moraes C.T. (2008). The mitochondrial impairment, oxidative stress and neurodegeneration connection: Reality or just an attractive hypothesis?. Trends Neurosci..

[20] Munné-Bosch S., Alegre L. (2002). Plant aging increases oxidative stress in chloroplasts. Planta.

[21] Islam R.A., Rallis C. (2023). Ribosomal biogenesis and heterogeneity in development, disease, and aging. Epigenomes.

[22] Macintosh K.B., Bonham-Smith P.C. (2006). Ribosomal protein gene regulation: What about plants?. Can. J. Bot..

[23] Whittle C.A., Krochko J.E. (2009). Transcript profiling provides evidence of functional divergence and expression networks among ribosomal protein gene paralogs in *Brassica napus*. Plant Cell.

[24] Delorme-Hinoux V., Mbodj A., Brando S., De Bures A., Llauro C., Covato F., Garrigue J., Guisset C., Borrut J., Mirouze M. (2023). 45S rDNA diversity in natura as one step towards ribosomal heterogeneity in *Arabidopsis thaliana*. Plants.

[25] Martinez-Seidel F., Beine-Golovchuk O., Hsieh Y.C., Kopka J. (2020). Systematic review of plant ribosome heterogeneity and specialization. Front. Plant Sci..

[26] Rosado A., Li R., van de Ven W., Hsu E., Raikhel N.V. (2012). Arabidopsis ribosomal proteins control developmental programs through translational regulation of auxin response factors. Proc. Natl. Acad. Sci. USA.

[27] Böttger E.C., Santhosh Kumar H., Steiner A., Sotirakis E., Thiam K., Isnard Petit P., Seebeck P., Wolfer D.P., Shcherbakov D., Akbergenov R. (2025). Translational error in mice increases with ageing in an organ-dependent manner. Nat. Commun..

[28] Steffen K.K., MacKay V.L., Kerr E.O., Tsuchiya M., Hu D., Fox L.A., Dang N., Johnston E.D., Oakes J.A., Tchao B.N. (2008). Yeast life span extension by depletion of 60s ribosomal subunits is mediated by Gcn4. Cell.

[29] Endo Y., Mitsui K., Motizuki M., Tsurugi K. (1987). The mechanism of action of ricin and related toxic lectins on eukaryotic ribosomes. The site and the characteristics of the modification in 28S ribosomal RNA caused by the toxins. J. Biol. Chem..

[30] Stirpe F. (2004). Ribosome-inactivating proteins. Toxicon.

[31] Kast A., Klassen R., Meinhardt F. (2014). rRNA fragmentation induced by a yeast killer toxin. Mol. Microbiol..

[32] Zhigailov A., Akbergenov R., Polimbetova N. (2022). Study of the role of RNA-interference in the regulation of discrete fragmentation of 18S rRNA in plants. Exp. Biol..

[33] Zhigailov A.V., Nizkorodova A.S., Sharipov K.O., Polimbetova N.S., Iskakov B.K. (2023). Glyphosate treatment mediates the accumulation of small discrete 5’- and 3’-terminal fragments of 18S rRNA in plant cells. Vavilovskii Zhurnal Genet. Sel..

[34] Zhigailov A.V., Babaylova E.S., Polimbetova N.S., Graifer D.M., Karpova G.G., Iskakov B.K. (2011). Putative implication of 3′-terminal segment of 18S rRNA in translation initiation of uncapped mRNAs in plants. Mol. Biol..

[35] Chen Z., Sun Y., Yang X., Wu Z., Guo K., Niu X., Wang Q., Ruan J., Bu W., Gao S. (2017). Two featured series of rRNA-derived RNA fragments (rRFs) constitute a novel class of small RNAs. PLoS ONE.

[36] Henras A.K., Plisson-Chastang C., O’Donohue M.F., Chakraborty A., Gleizes P.E. (2015). An overview of pre-ribosomal RNA processing in eukaryotes. Wiley Interdiscip. Rev. RNA.

[37] Lin J., Xu K., Roth J.A., Ji L. (2016). Detection of siRNA-mediated target mRNA cleavage activities in human cells by a novel stem-loop array RT-PCR analysis. Biochem. Biophys. Rep..

[38] Sayers E.W., Cavanaugh M., Frisse L., Pruitt K.D., Schneider V.A., Underwood B.A., Yankie L., Karsch-Mizrachi I. (2025). GenBank 2025 update. Nucl. Acids Res..

[39] Barrs H.D., Weatherley P.E. (1962). A re-examination of the relative turgidity techniques for estimating water deficits in leaves. Aust. J. Biol. Sci..

[40] Hoagland D.R., Arnon D.I. (1950). The water-culture method for growing plants without soil. Calif. Agric. Exp. Stn. Circ..

[41] Smirnova J., Loerke J., Kleinau G., Schmidt A., Bürger J., Meyer E.H., Mielke T., Scheerer P., Bock R., Spahn C.M.T. (2023). Structure of the actively translating plant 80S ribosome at 2.2 Å resolution. Nat. Plants.

[42] Cannone J.J., Subramanian S., Schnare M.N., Collett J.R., D’Souza L.M., Du Y., Feng B., Lin N., Madabusi L.V., Müller K.M. (2002). The comparative RNA web (CRW) site: An online database of comparative sequence and structure information for ribosomal, intron, and other RNAs. BMC Bioinform..

[43] Fu H., Yang Y. (2023). How Plants Tolerate Salt Stress. Curr. Issues Mol. Biol..

[44] Baimagambetova K.K., Abugaliev S.G. (2012). Prospects for variety selection of spring wheat Open Company “the Kazakh scientific research institute of agriculture and plant growing” JSC “KazAgroInnovation” MSH RK. Kazn. Bull. Biol. Ser..

[45] Joyce S.M., Cassells A.C., Mohan Jain S. (2003). Stress and aberrant phenotypes *in vitro* culture. Plant Cell Tissue Organ Cult..

[46] Smailov S.K., Lee A.V., Iskakov B.K. (1993). Study of phosphorylation of translation elongation factor 2 (EF-2) from wheat germ. FEBS Lett..

[47] Gallie D.R., Le H., Caldwell C., Browning K.S. (1998). Analysis of translation elongation factors from wheat during development and following heat shock. Biochem. Biophys. Res. Comm..

[48] Echevarria-Zomeno S., Yanguez E., Fernandez-Bautista N., Castro Sanz A.B., Ferrando A., Castellano M.M. (2013). Regulation of translation initiation under biotic and abiotic stresses. Int. J. Mol. Sci..

[49] Browning K.S., Bailey-Serres J. (2015). Mechanism of cytoplasmic mRNA translation. Arab. Book.

[50] Baird T.D., Wek R.C. (2012). Eukaryotic initiation factor 2 phosphorylation and translational control in metabolism. Adv. Nutr..

[51] Shaikhin S.M., Smailov S.K., Lee A.V., Kozhanov E.V., Iskakov B.K. (1992). Interaction of wheat germ translation initiation factor 2 with GDP and GTP. Biochimie.

[52] Zhigailov A.V., Alexandrova A.M., Nizkorodova A.S., Stanbekova G.E., Kryldakov R.V., Karpova O.V., Polimbetova N.S., Halford N.G., Iskakov B.K. (2020). Evidence that Phosphorylation of the αsubunit of eIF2 does not essentially inhibit mRNA translation in wheat germ cell-free system. Front. Plant Sci..

[53] Immanuel T.M., Greenwood D.R., MacDiarmid R.M. (2012). A critical review of translation initiation factor eIF2α kinases in plants—regulating protein synthesis during stress. Funct. Plant Biol..

[54] Athar H.U., Zulfiqar F., Moosa A., Ashraf M., Zafar Z.U., Zhang L., Ahmed N., Kalaji H.M., Nafees M., Hossain M.A. (2022). Salt stress proteins in plants: An overview. Front. Plant Sci..

[55] Rhodes P.R., Matsuda K. (1976). Water stress, rapid polyribosome reductions and growth. Plant Physiol..

[56] Kawaguchi R., Girke T., Bray E.A., Bailey-Serres J. (2004). Differential mRNA translation contributes to gene regulation under non-stress and dehydration stress conditions in *Arabidopsis thaliana*. Plant J..

[57] Rook F., Gerrits N., Kortstee A., van Kampen M., Borrias M., Weisbeek P., Smeekens S. (1998). Sucrose-specific signalling represses translation of the *Arabidopsis* ATB2 bZIP transcription factor gene. Plant J..

[58] Muñoz A., Castellano M.M. (2012). Regulation of translation initiation under abiotic stress conditions in plants: Is it a conserved or not so conserved process among eukaryotes?. Comp. Funct. Genom..

[59] Zhang Y., Dickinson J.R., Paul M.J., Halford N.G. (2003). Molecular cloning of an arabidopsis homologue of GCN2, a protein kinase involved in co-ordinated response to amino acid starvation. Planta.

[60] Inada T., Beckmann R. (2024). Mechanisms of Translation-coupled Quality Control. J. Mol. Biol..

[61] LaRiviere F.J., Cole S.E., Ferullo D.J., Moore M.J. (2006). A late-acting quality control process for mature eukaryotic rRNAs. Mol. Cell.

