# Supplementary material for: The Content of Small 18S rRNA Fragments Is Regulated Developmentally and in Response to Stress in Plants
Source: Plants (Basel). 2026 May 15;15(10):1512. doi: 10.3390/plants15101512 (PMC13210519; doi:10.3390/plants15101512)
Supplement: Supplementary file 1 [file plants-15-01512-s001.zip › plants-4274504-Supplementary-S1.pdf]

Supplementary Table S1. Primers used in the SLA-RT-PCR

Primer/ Name	Primer Sequence (5′ → 3′)[a]	Orien-tation	Target posi-tion[b]
5(75)-RT-1	GTCGAATTCAGTGCGGGTCCGAGGTATTCGCAC-<br>TGAATTCGACATTGGT	R	69-74
5(75)-RT-2	GTCGAATTCAGTGCGGGTCCGAGGTATTCGCAC-<br>TGAATTCGACAATTGG	R	70-75
5(75)-RT-3	GTCGAATTCAGTGCGGGTCCGAGGTATTCGCAC-<br>TGAATTCGACAAATTG	R	71-76
5(75)-RT-4	GTCGAATTCAGTGCGGGTCCGAGGTATTCGCACTGAATTCGAC-<br>CAAATT	R	72-77
5(75)-RT-5	GTCGAATTCAGTGCGGGTCCGAGGTATTCGCAC-<br>TGAATTCGACTCAAAT	R	73-78
5(75)-RT-6	GTCGAATTCAGTGCGGGTCCGAGGTATTCGCAC-<br>TGAATTCGACTTCAAA	R	74-79
5(75)-RT-7	GTCGAATTCAGTGCGGGTCCGAGGTATTCGCACTGAATTCGAC-<br>GTTCAA	R	75-80
5(75)-RT-8	GTCGAATTCAGTGCGGGTCCGAGGTATTCGCAC-<br>TGAATTCGACAGTTCA	R	76-81
5(75)-RT-9	GTCGAATTCAGTGCGGGTCCGAGGTATTCGCACTGAATTCGAC-<br>CAGTTC	R	77-82
5(75)-RT-10	GTCGAATTCAGTGCGGGTCCGAGGTATTCGCAC-<br>TGAATTCGACACAGTT	R	78-83
5(75)-RT-11	GTCGAATTCAGTGCGGGTCCGAGGTATTCGCACTGAATTCGAC-<br>CACAGT	R	79-84
5(75)-RT-12	GTCGAATTCAGTGCGGGTCCGAGGTATTCGCAC-<br>TGAATTCGACTCACAG	R	80-85
5(75)-RT-13	GTCGAATTCAGTGCGGGTCCGAGGTATTCGCAC-<br>TGAATTCGACTTCACA	R	81-86
5(75)-RT-14	GTCGAATTCAGTGCGGGTCCGAGGTATTCGCAC-<br>TGAATTCGACTTTCAC	R	82-87
5(75)-RT-15	GTCGAATTCAGTGCGGGTCCGAGGTATTCGCACTGAATTCGAC-<br>GTTTCA	R	83-88
5(132)-RT-1	GTCGAATTCAGTGCGGGTCCGAGGTATTCGCAC-<br>TGAATTCGACTCAAAC	R	120-125
5(132)-RT-2	GTCGAATTCAGTGCGGGTCCGAGGTATTCGCAC-<br>TGAATTCGACATCAAA	R	121-126
5(132)-RT-3	GTCGAATTCAGTGCGGGTCCGAGGTATTCGCACTGAATTCGAC-<br>CATCAA	R	122-127
5(132)-RT-4	GTCGAATTCAGTGCGGGTCCGAGGTATTCGCAC-<br>TGAATTCGACCCATCA	R	123-128
5(132)-RT-5	GTCGAATTCAGTGCGGGTCCGAGGTATTCGCAC-<br>TGAATTCGACACCATC	R	124-129
5(132)-RT-6	GTCGAATTCAGTGCGGGTCCGAGGTATTCGCACTGAATTCGAC-<br>TACCAT	R	125-130
5(132)-RT-7	GTCGAATTCAGTGCGGGTCCGAGGTATTCGCACTGAATTCGAC-<br>GTACCA	R	126-131
5(132)-RT-8	GTCGAATTCAGTGCGGGTCCGAGGTATTCGCACTGAATTCGAC-<br>CGTACC	R	127-132

5(132)-RT-9	GTCGAATTCAGTGCGGGTCCGAGGTATTCGCAC-TGAATTCGACACGTAC	R	128-133
5(132)-RT-10	GTCGAATTCAGTGCGGGTCCGAGGTATTCGCACTGAATTCGAC-CACGTA	R	129-134
5(132)-RT-11	GTCGAATTCAGTGCGGGTCCGAGGTATTCGCACTGAATTCGAC-GCACGT	R	130-135
5(132)-RT-12	GTCGAATTCAGTGCGGGTCCGAGGTATTCGCAC-TGAATTCGACAGCACG	R	131-136
5(132)-RT-13	GTCGAATTCAGTGCGGGTCCGAGGTATTCGCAC-TGAATTCGACTAGCAC	R	132-137
5(132)-RT-14	GTCGAATTCAGTGCGGGTCCGAGGTATTCGCACTGAATTCGAC-GTAGCA	R	133-138
5(132)-RT-15	GTCGAATTCAGTGCGGGTCCGAGGTATTCGCAC-TGAATTCGACAGTAGC	R	134-139
3(110)-RT-1	GTCGAATTCAGTGCGGGTCCGAGGTATTCGCACTGAATTCGAC-GCCGCG	R	1691-1696
3(110)-RT-2	GTCGAATTCAGTGCGGGTCCGAGGTATTCGCACTGAATTCGAC-CGCCGC	R	1692-1697
3(110)-RT-3	GTCGAATTCAGTGCGGGTCCGAGGTATTCGCAC-TGAATTCGACTCGCCG	R	1693-1698
3(110)-RT-4	GTCGAATTCAGTGCGGGTCCGAGGTATTCGCACTGAATTCGAC-GTCGCC	R	1694-1699
3(110)-RT-5	GTCGAATTCAGTGCGGGTCCGAGGTATTCGCACTGAATTCGAC-CGTCGC	R	1695-1700
3(110)-RT-6	GTCGAATTCAGTGCGGGTCCGAGGTATTCGCAC-TGAATTCGACCCGTCG	R	1696-1701
3(110)-RT-7	GTCGAATTCAGTGCGGGTCCGAGGTATTCGCAC-TGAATTCGACCCCGTC	R	1697-1702
3(110)-RT-8	GTCGAATTCAGTGCGGGTCCGAGGTATTCGCAC-TGAATTCGACCCCCGT	R	1698-1703
3(110)-RT-9	GTCGAATTCAGTGCGGGTCCGAGGTATTCGCAC-TGAATTCGACCCCCCG	R	1699-1704
3(110)-RT-10	GTCGAATTCAGTGCGGGTCCGAGGTATTCGCACTGAATTCGAC-GCCCCC	R	1700-1705
3(110)-RT-11	GTCGAATTCAGTGCGGGTCCGAGGTATTCGCACTGAATTCGAC-CGCCCC	R	1701-1706
3(110)-RT-12	GTCGAATTCAGTGCGGGTCCGAGGTATTCGCAC-TGAATTCGACCCGCCC	R	1702-1707
3(110)-RT-13	GTCGAATTCAGTGCGGGTCCGAGGTATTCGCAC-TGAATTCGACACCGCC	R	1703-1708
3(110)-RT-14	GTCGAATTCAGTGCGGGTCCGAGGTATTCGCAC-TGAATTCGACAACCGC	R	1704-1709
3(110)-RT-15	GTCGAATTCAGTGCGGGTCCGAGGTATTCGCACTGAATTCGAC-GAACCG	R	1705-1710
SLA-PCR-UniRev	GTGCGGGTCCGAGGTATTC	R	-
5(75)-1-F	TACCTGGTTGATCCTGCCAG	F	1-20
3(110)-1434-F	AGGTCTGTCATGCCCTTAGA	F	1434-1453
Ctrl-166-R	TAGCTCTAGAATTACTACGGTT	R	145-166
Ctrl-1749-R	ATAAGGTTCAATGGACTTCTC	R	1729-1749
3(110)-1434-Acc-F	TTAGGTACCAGGTCTGTCATGCCCTTAGA	F	1434-1453
3(110)-1698-ER-R	AATGAATTCGCCGCGATCCGAACACT	R	1681-1698

[a] Nucleotides complementary to specific regions of *Triticum aestivum* 18S rRNA are underlined. [b] *Triticum aestivum* L. 18S rRNA gene (GenBank: AY049040).
