# Supplementary material for: The Content of Small 18S rRNA Fragments Is Regulated Developmentally and in Response to Stress in Plants
Source: Plants (Basel). 2026 May 15;15(10):1512. doi: 10.3390/plants15101512 (PMC13210519; doi:10.3390/plants15101512)
Supplement: Supplementary file 1 [file plants-15-01512-s001.zip › plants-4274504-Supplementary-S3.pdf]

plants

Supplementary Material S3. Validation of SLA-RT-PCR method for mapping disruption sites of plant 18S rRNA molecules

S.1. Generation the Ta18S-3′end-1698/1699 control RNA and validation of the SLA-RT-PCR method

S.1.1 *Triticum aestivum* 18S ribosomal RNA (GenBank: AY049040). G1698 is marked in yellow, while A1699 is marked in blue.

```
(5')TACCUGGUUGAUCCUGCCAGUAGUCAUAUGCUUGUCUCAAAGAUUAAGCCAUGCAUGUGCAAGUAUGAACCAA
UUUGAACUGUGAAACUGCGAAUGGCUCAUUAAAUCAGUUAUAGUUUGUUUGAUGGUACGUGCUACUCGGAUAACCGU
AGUAAUUCUAGAGCUAAUACGUGCAACAAACCCCGACUUCUGGGAGGGGCGCAUUUAUUAGAUAAAAGGCUGACGCG
GGCUCUGCUCGCUGAUCCGAUGAUUCAUGAUAACUCGACGGAUCGCACGGCCUUCGUGCCGGCGACGCAUCAUUCAA
AUUUCUGCCCUAUCAACUUUCGAUGGUAGGAUAGGGGCCUACCAUGGUGGUGACGGGUGACGGAGAAUUAGGGUUCG
AUUCCGGAGAGGGAGCCUGAGAAACGGCUACCACAUCCAAGGAAGGCAGCAGGCGCGCAAAUUACCCAAUCCUGACA
CGGGGAGGUAGUGACAAUAAAUAACAAUACCGGGCGCAUUAGUGUCUGGUAAUUGGAAUGAGUACAAUCUAAAUCCC
UUAACGAGGAUCCAUUGGAGGGCAAGUCUGGUGCCAGCAGCCGCGGUAAUUCCAGCUCCAAUAGCGUAUAUUUAAGU
UGUUGCAGUUAAAAAGCUCGUAGUUGGACCUUGGGCCGGGUCGGCCGGUCCGCCUCACGGCGAGCACCGACCUACUC
GACCCUUCGGCCGGCAUCGCGCUCCUAGCCUUAAUUGGCCGGGUCGUGUUUCCGGCAUCGUUACUUUGAAGAAAUUA
GAGUGCUCAAAGCAAGCCAUCGCUCUGGAUACAUUAGCAUGGGAUAACAUCAUAGGAUUCCGGUCCUAUUGUGUUGG
CCUUCGGGAUCGGAGUAAUGAUUAAUAGGGACAGUCGGGGGCAUUCGUAUUUCAUAGUCAGAGGUGAAAUUCUUGGA
UUUAUGAAAGACGAACAACUGCGAAAGCAUUUGCCAAGGAUGUUUUCAUUAAUCAAGAACGAAAGUUGGGGGCUCGA
AGACGAUCAGAUACCGUCCUAGUCUCAACCAUAAACGAUGCCGACCAGGGAUCGGCGGAUGUUGCUUAUAGGACUCC
GCCGGCACCUUAUGAGAAAUCAAAGUCUUUGGGUUCCGGGGGGAGUAUGGUCGCAAGGCUGAAACUUAAAGGAAUUG
ACGGAAGGGCACCACCAGGCGUGGAGCCUGCAGCUUAAUUUGACUCAACACGGGGAAACUUACCAGGUCCAGACAUA
GCAAGGAUUGACAGACUGAGAGCUCUUUCUUGAUUCUAUGGGUGGUGGUGCAUGGCCGUUCUUAGUUGGUGGAGCGA
UUUGUCUGGUUAAUUCCGUUAACGAACGAGACCUCAGCCUGCUAACUAGCUAUGCGGAGCCAUCCCUCCGCAGCUAG
CUUCUUAGAGGGACUAUGGCCGUUUAGGCCACGGAAGUUUGAGGCAAUAACAGGUCUGUCAUGCCCUUAGAUGUUCU
GGGCCGCACGCGCGCUACACUGAUGUAUUCAACGAGUAUAUAGCCUUGGCCGACAGGCCCGGGUAAUCUUGGGAAAU
UUCAUCGUGAUGGGGAUAGAUCAUUGCAAUUGUUGGUCUUCAACGAGGAAUGCCUAGUAAGCGCGAGUCAUCAGCUC
GCGUUGACUACGUCCCUGCCCUUUGUACACACCGCCCGUCGCUCCUACCGAUUGAAUGGUCCGGUGAAGUGUUCGGA
UCGCGGCGACGGGGGCGGUUCGCCGCCCCCGACGUCGCGAGAAGUCCAUUGAACCUUAUCAUUUAGAGGAAGCAGAA
GUCGUAACAAGGUUUCCGUAGGUGAACCUGCGGAAGGAUCAUUG
```

S.1.2 The 316-bp control RT-PCR product '3(100)-Ctrl' (last line (17th) on the Figure 2,c): This is the positive control for 3′-terminal 18S rRNA fragments, utilizing wheat RNA, the primer Ctrl-1749-R (5′-ATAAGGTTCAATGGACTTCTC) for reverse transcription, and primers 3(110)-1434-F (5′-AGGTCTGTCATGCCCTTAGA) and Ctrl-1749-R (5′-ATAAGGTTCAATGGACTTCTC) for PCR:

```
AGGTCTGTCATGCCCTTAGATGTTCTGGGCCGCACGCGCGCTACACTGATGTATTCAACGAGTATATAGCCTTGGCC
GACAGGCCCGGGTAATCTTGGGAAATTTCATCGTGATGGGGATAGATCATTGCAATTGTTGGTCTTCAACGAGGAAT
GCCTAGTAAGCGCGAGTCATCAGCTCGCGTTGACTACGTCCCTGCCCTTTGTACACACCGCCCGTCGCTCCTACCGA
TTGAATGGTCCGGTGAAGTGTTCGGATCGCGGCGACGGGGGCGGTTCGCCGCCCCCGACGTCGCGAGAAGTCCATTG
AACCTTAT
```

S.1.3. Disruption of *T. aestivum* 18S rRNA at 1698/1699 site:

```
(5')UACCUGGUUGAUCCUGCCAGUAGUCAUAUGCUUGUCUCAAAGAUUAAGCCAUGCAUGUGCAAGUAUGAACCAA
UUUGAACUGUGAAACUGCGAAUGGCUCAUUAAAUCAGUUAUAGUUUGUUUGAUGGUACGUGCUACUCGGAUAACCGU
AGUAAUUCUAGAGCUAAUACGUGCAACAAACCCCGACUUCUGGGAGGGGCGCAUUUAUUAGAUAAAAGGCUGACGCG
GGCUCUGCUCGCUGAUCCGAUGAUUCAUGAUAACUCGACGGAUCGCACGGCCUUCGUGCCGGCGACGCAUCAUUCAA
AUUUCUGCCCUAUCAACUUUCGAUGGUAGGAUAGGGGCCUACCAUGGUGGUGACGGGUGACGGAGAAUUAGGGUUCG
AUUCCGGAGAGGGAGCCUGAGAAACGGCUACCACAUCCAAGGAAGGCAGCAGGCGCGCAAAUUACCCAAUCCUGACA
CGGGGAGGUAGUGACAAUAAAUAACAAUACCGGGCGCAUUAGUGUCUGGUAAUUGGAAUGAGUACAAUCUAAAUCCC
UUAACGAGGAUCCAUUGGAGGGCAAGUCUGGUGCCAGCAGCCGCGGUAAUUCCAGCUCCAAUAGCGUAUAUUUAAGU
UGUUGCAGUUAAAAAGCUCGUAGUUGGACCUUGGGCCGGGUCGGCCGGUCCGCCUCACGGCGAGCACCGACCUACUC
GACCCUUCGGCCGGCAUCGCGCUCCUAGCCUUAAUUGGCCGGGUCGUGUUUCCGGCAUCGUUACUUUGAAGAAAUUA
GAGUGCUCAAAGCAAGCCAUCGCUCUGGAUACAUUAGCAUGGGAUAACAUCAUAGGAUUCCGGUCCUAUUGUGUUGG
CCUUCGGGAUCGGAGUAAUGAUUAAUAGGGACAGUCGGGGGCAUUCGUAUUUCAUAGUCAGAGGUGAAAUUCUUGGA
UUUAUGAAAGACGAACAACUGCGAAAGCAUUUGCCAAGGAUGUUUUCAUUAAUCAAGAACGAAAGUUGGGGGCUCGA
AGACGAUCAGAUACCGUCCUAGUCUCAACCAUAAACGAUGCCGACCAGGGAUCGGCGGAUGUUGCUUAUAGGACUCC
GCCGGCACCUUAUGAGAAAUCAAAGUCUUUGGGUUCCGGGGGGAGUAUGGUCGCAAGGCUGAAACUUAAAGGAAUUG
ACGGAAGGGCACCACCAGGCGUGGAGCCUGCAGCUUAAUUUGACUCAACACGGGGAAACUUACCAGGUCCAGACAUA
GCAAGGAUUGACAGACUGAGAGCUCUUUCUUGAUUCUAUGGGUGGUGGUGCAUGGCCGUUCUUAGUUGGUGGAGCGA
UUUGUCUGGUUAAUUCCGUUAACGAACGAGACCUCAGCCUGCUAACUAGCUAUGCGGAGCCAUCCCUCCGCAGCUAG
CUUCUUAGAGGGACUAUGGCCGUUUAGGCCACGGAAGUUUGAGGCAAUAACAGGUCUGUCAUGCCCUUAGAUGUUCU
GGGCCGCACGCGCGCUACACUGAUGUAUUCAACGAGUAUAUAGCCUUGGCCGACAGGCCCGGGUAAUCUUGGGAAAU
UUCAUCGUGAUGGGGAUAGAUCAUUGCAAUUGUUGGUCUUCAACGAGGAAUGCCUAGUAAGCGCGAGUCAUCAGCUC
GCGUUGACUACGUCCCUGCCCUUUGUACACACCGCCCGUCGCUCCUACCGAUUGAAUGGUCCGGUGAAGUGUUCGGA
UCGCGGCG
```

```
(5')ACGGGGGCGGUUCGCCGCCCCCGACGUCGCGAGAAGUCCAUUGAACCUUAUCAUUUAGAGGAAGCAGAAGUCG
UAACAAGGUUUCCGUAGGUGAACCUGCGGAAGGAUCAUUG
```

S.1.4 The 1698-nt fragment of the *T. aestivum* 18S rRNA + SLA-RT primer '3(110)-RT-2' (5'-GTCGAATTCAGTGCGGTCCGAGGTATTCGCACTGAATTCGACCGCCGC):

```
(5')TACCTGGTTGATCCTGCCAGTAGTCATATGCTTGTCTCAAAGATTAAGCCATGCATGTGCAAGTATGAACCAA
TTTGAACTGTGAAACTGCGAATGGCTCATTAAATCAGTTATAGTTTGTTTGATGGTACGTGCTACTCGGATAACCGT
AGTAATTCTAGAGCTAATACGTGCAACAAACCCCGACTTCTGGGAGGGGCGCATTTATTAGATAAAAGGCTGACGCG
GGCTCTGCTCGCTGATCCGATGATTCATGATAACTCGACGGATCGCACGGCCTTCGTGCCGGCGACGCATCATTCAA
ATTTCTGCCCTATCAACTTTCGATGGTAGGATAGGGGCCTACCATGGTGGTGACGGGTGACGGAGAATTAGGGTTCG
ATTCCGGAGAGGGAGCCTGAGAAACGGCTACCACATCCAAGGAAGGCAGCAGGCGCGCAAATTACCCAATCCTGACA
CGGGGAGGTAGTGACAATAAATAACAATACCGGGCGCATTAGTGTCTGGTAATTGGAATGAGTACAATCTAAATCCC
TTAACGAGGATCCATTGGAGGGCAAGTCTGGTGCCAGCAGCCGCGGTAATTCCAGCTCCAATAGCGTATATTTAAGT
TGTTGCAGTTAAAAAGCTCGTAGTTGGACCTTGGGCCGGGTCGGCCGGTCCGCCTCACGGCGAGCACCGACCTACTC
GACCCTTCGGCCGGCATCGCGCTCCTAGCCTTAATTGGCCGGGTCGTGTTTCCGGCATCGTTACTTTGAAGAAATTA
GAGTGCTCAAAGCAAGCCATCGCTCTGGATACATTAGCATGGGATAACATCATAGGATTCCGGTCCTATTGTGTTGG
CCTTCGGGATCGGAGTAATGATTAATAGGGACAGTCGGGGGCATTCGTATTTCATAGTCAGAGGTGAAATTCTTGGA
TTTATGAAAGACGAACAACTGCGAAAGCATTTGCCAAGGATGTTTTCATTAATCAAGAACGAAAGTTGGGGGCTCGA
AGACGATCAGATACCGTCCTAGTCTCAACCATAAACGATGCCGACCAGGGATCGGCGGATGTTGCTTATAGGACTCC
GCCGGCACCTTATGAGAAATCAAAGTCTTTGGGTTCCGGGGGGAGTATGGTCGCAAGGCTGAAACTTAAAGGAATTG
ACGGAAGGGCACCACCAGGCGTGGAGCCTGCAGCTTAATTTGACTCAACACGGGGAAACTTACCAGGTCCAGACATA
GCAAGGATTGACAGACTGAGAGCTCTTTCTTGATTCTATGGGTGGTGGTGCATGGCCGTTCTTAGTTGGTGGAGCGA
TTTGTCTGGTTAATTCCGTTAACGAACGAGACCTCAGCCTGCTAACTAGCTATGCGGAGCCATCCCTCCGCAGCTAG
CTTCTTAGAGGGACTATGGCCGTTTAGGCCACGGAAGTTTGAGGCAATAACAGGTCTGTCATGCCCTTAGATGTTCT
GGGCCGCACGCGCGCTACACTGATGTATTCAACGAGTATATAGCCTTGGCCGACAGGCCCGGGTAATCTTGGGAAAT
TTCATCGTGATGGGGATAGATCATTGCAATTGTTGGTCTTCAACGAGGAATGCCTAGTAAGCGCGAGTCATCAGCTC
GCGTTGACTACGTCCCTGCCCTTTGTACACACCGCCCGTCGCTCCTACCGATTGAATGGTCCGGTGAAGTGTTCGGA
TCGCGGCG
                        GTC
                       G    C
        GTCGAATTCAGTGCG      G
3'CGCCGCCAGCTTAAGTCACGC      A   '3(110)-RT-2' SLA reverse primer
                       T    G
                        TATG
```

S.1.5 The resulting cDNA synthesized from *T. aestivum* L total RNA using the reverse SLA-RT primer '3(110)-RT-2' (5′-GTCGAATTCAGTGCGGGTCCGAGGTATTCGCAC-TGAATTCGACCGCCGC) targeting the 1692-1697 nt region of the *T. aestivum* L. 18S rRNA gene (GenBank: AY049040):

(5′)GTCGAATTCAGTGCGGGTCCGAGGTATTCGCACTGAATTCGACCGCCGCGATCCGAACACTTCACCGGACCAT
TCAATCGGTAGGAGCGACGGGCGGTGTGTACAAAGGGCAGGGACGTAGTCAACGCGAGCTGATGACTCGCGCTTACT
AGGCATTCCTCGTTGAAGACCAACAATTGCAATGATCTATCCCCATCACGATGAAATTTCCCAAGATTACCCGGGCC
TGTCGGCCAAGGCTATATACTCGTTGAATACATCAGTGTAGCGCGCGTGCGGCCCAGAACATCTAAGGGCATGACAG
ACCTGTTATTGCCTCAAACTTCCGTGGCCTAAACGGCCATAGTCCCTCTAAGAAGCTAGCTGCGGAGGGATGGCTCC
GCATAGCTAGTTAGCAGGCTGAGGTCTCGTTCGTTAACGGAATTAACCAGACAAATCGCTCCACCAACTAAGAACGG
CCATGCACCACCACCCATAGAATCAAGAAAGAGCTCTCAGTCTGTCAATCCTTGCTATGTCTGGACCTGGTAAGTTT
CCCCGTGTTGAGTCAAATTAAGCTGCAGGCTCCACGCCTGGTGGTGCCCTTCCGTCAATTCCTTTAAGTTTCAGCCT
TGCGACCATACTCCCCCCGGAACCCAAAGACTTTGATTTCTCATAAGGTGCCGGCGGAGTCCTATAAGCAACATCCG
CCGATCCCTGGTCGGCATCGTTTATGGTTGAGACTAGGACGGTATCTGATCGTCTTCGAGCCCCCAACTTTCGTTCT
TGATTAATGAAAACATCCTTGGCAAATGCTTTCGCAGTTGTTCGTCTTTCATAAATCCAAGAATTTCACCTCTGACT
ATGAAATACGAATGCCCCCGACTGTCCCTATTAATCATTACTCCGATCCCGAAGGCCAACACAATAGGACCGGAATC
CTATGATGTTATCCCATGCTAATGTATCCAGAGCGATGGCTTGCTTTGAGCACTCTAATTTCTTCAAAGTAACGATG
CCGGAAACACGACCCGGCCAATTAAGGCTAGGAGCGCGATGCCGGCCGAAGGGTCGAGTAGGTCGGTGCTCGCCGTG
AGGCGGACCGGCCGACCCGGCCCAAGGTCCAACTACGAGCTTTTTAACTGCAACAACTTAAATATACGCTATTGGAG
CTGGAATTACCGCGGCTGCTGGCACCAGACTTGCCCTCCAATGGATCCTCGTTAAGGGATTTAGATTGTACTCATTC
CAATTACCAGACACTAATGCGCCCGGTATTGTTATTTATTGTCACTACCTCCCCGTGTCAGGATTGGGTAATTTGCG
CGCCTGCTGCCTTCCTTGGATGTGGTAGCCGTTTCTCAGGCTCCCTCTCCGGAATCGAACCCTAATTCTCCGTCACC
CGTCACCACCATGGTAGGCCCCTATCCTACCATCGAAAGTTGATAGGGCAGAAATTTGAATGATGCGTCGCCGGCAC
GAAGGCCGTGCGATCCGTCGAGTTATCATGAATCATCGGATCAGCGAGCAGAGCCCGCGTCAGCCTTTTATCTAATA
AATGCGCCCCTCCCAGAAGTCGGGGTTTGTTGCACGTATTAGCTCTAGAATTACTACGGTTATCCGAGTAGCACGTA
CCATCAAACAAACTATAACTGATTTAATGAGCCATTCGCAGTTTCACAGTTCAAATTGGTTCATACTTGCACATGCA
TGGCTTAATCTTTGAGACAAGCATATGACTACTGGCAGGATCAACCAGGTA

S.1.6 The 298-bp RT-PCR product '3(110)-RT-2' (line 2 in Figure 2c) generated from cDNA using primers '3(110)-1434-F' (5′-AGGTCTGTCATGCCCTTAGA) and 'SLA-PCR-UniRev' (5′-GTGCGGGTCCGAGGTATTC):

AGGTCTGTCATGCCCTTAGATGTTCTGGGCCGCACGCGCGCTACACTGATGTATTCAACGAGTATATAGCCTTGGCC
GACAGGCCCGGGTAATCTTGGGAAATTTCATCGTGATGGGGATAGATCATTGCAATTGTTGGTCTTCAACGAGGAAT
GCCTAGTAAGCGCGAGTCATCAGCTCGCGTTGACTACGTCCCTGCCCTTTGTACACACCGCCCGTCGCTCCTACCGA
TTGAATGGTCCGGTGAAGTGTTCGGATCGCGGCGGTCGAATTCAGTGCGAATACCTCGGACCCGCAC

S.1.7 Generation the Ta18S-3′end-1698/1699 control RNA for method validation:

Generation of the *Ta18S-3′end-1698/1699* RT-PCR product using wheat RNA as a template, primer Ctrl-1749-R (5′-ATAAGGTTCAATGGACTTCTC) for reverse transcription, and primers 3(110)-1434-Acc-F (5′-TAGGTACCAGGTCTGTCATGCCCTTAGA) and 3(110)-1698-ER-R (5′-AATGAATTCGCCGCGATCCGAACACT) for PCR:

TTA*GGTACC*AGGTCTGTCATGCCCTTAGATGTTCTGGGCCGCACGCGCGCTACACTGATGTATTCAACGAGTATATA
GCCTTGGCCGACAGGCCCGGGTAATCTTGGGAAATTTCATCGTGATGGGGATAGATCATTGCAATTGTTGGTCTTCA
ACGAGGAATGCCTAGTAAGCGCGAGTCATCAGCTCGCGTTGACTACGTCCCTGCCCTTTGTACACACCGCCCGTCGC
TCCTACCGATTGAATGGTCCGGTGAAGTGTTCGGATCGCGGC*GAATTC*TAA

The resulting fragment was then digested with the restriction endonucleases *Acc65*I and *EcoR*I and cloned into the corresponding sites of the pBluescript KSII(+) vector, resulting in the generation of the *Ta18S-3′end-1698/1699-pBKS* DNA construct. A schematic representation of this construct is shown in Figure S.1.

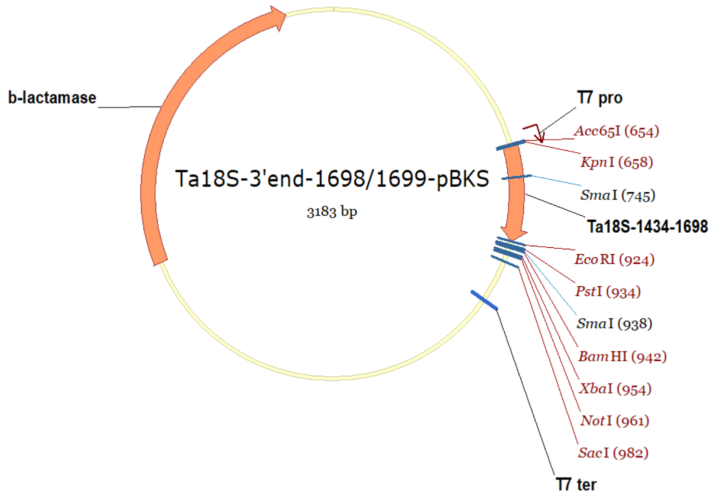

Figure S.1. The schematic representation of the *Ta18S-3'end-1698/1699-pBKS* DNA-construct.

The *Ta18S-3'end-1698/1699-pBKS* construct was linearized with *Eco*RI and subsequently used as a template for in vitro transcription of the Ta18S-3'end-1698/1699 control RNA. Transcription was carried out using T7 RNA polymerase (Thermo Fisher Scientific, Waltham, MA, USA) according to the manufacturer's protocol. Finally, the synthesized RNA was treated with DNase I (New England Biolabs, USA), precipitated with 3 M LiCl (Sigma-Aldrich, St. Louis, MO, USA), and used for SLA-RT-PCR.

S.1.8 The SLA-RT-PCR was performed according to the protocol for mapping 18S rRNA cleavage sites within the 1698–1712 region. The results are shown in Figure S.2.

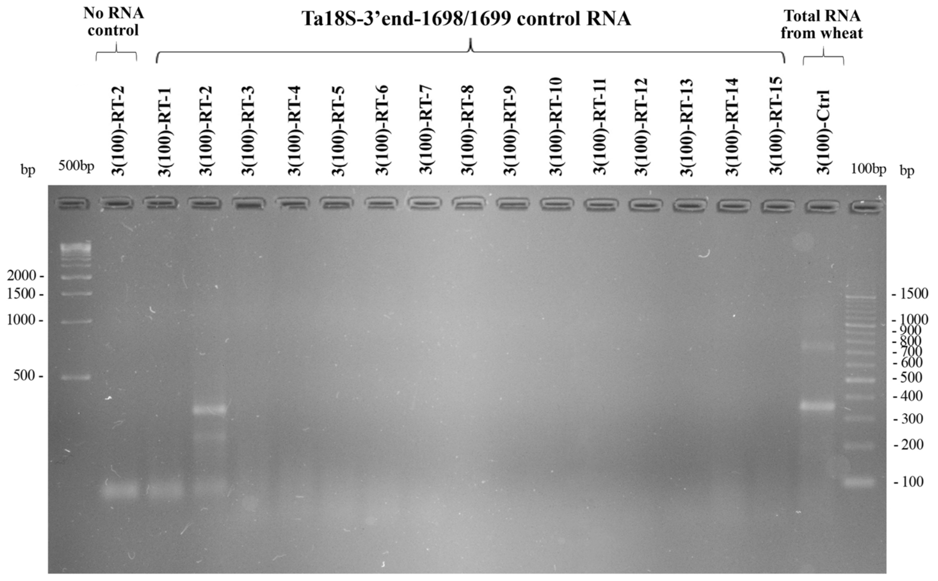

Figure S.2. Electrophoretic analysis of SLA-RT-PCR products. A 1.5% agarose gel is shown. The primers used for reverse transcription are indicated above the lanes. Lanes 1–16: PCR amplification using primers 3(110)-1434-F / SLA-PCR-UniRev. The 100 bp and 500 bp DNA ladders are included for reference. '3(100)-Ctrl': This is the positive control for 3'-terminal 18S rRNA fragments, utilizing wheat RNA, the primer Ctrl-1749-R for reverse transcription, and primers 3(110)-1434-F and Ctrl-1749-R for PCR. The primer sequences can be found in Supplementary Material S1.

S.2. Negative controls for SLA-RT-PCR validation (related to Figure 2)

S.2.1 Reactions without reverse transcriptase

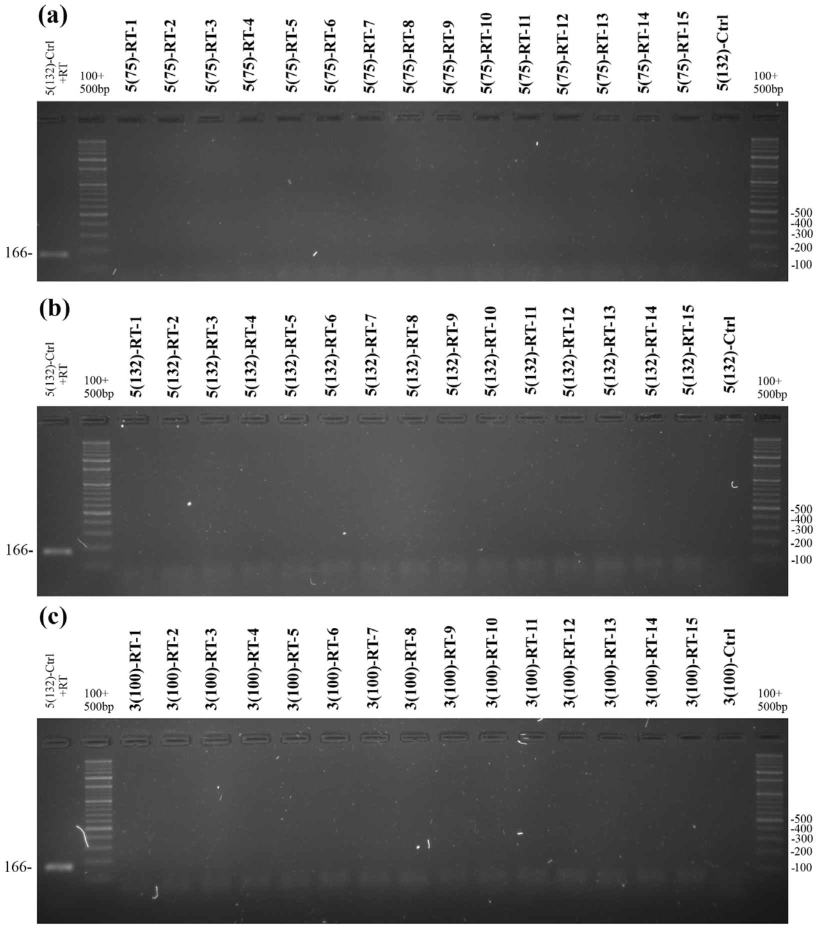

Figure S.3. Electrophoretic analysis of SLA-RT-PCR amplification products (reactions without reverse transcriptase). The 1.5% agarose gels are shown (each panel represents a specific target region of the 18S rRNA). **(a)** Testing of the 75–89 region (using primers 5(75)-1-F / SLA-PCR-UniRev for PCR); **(b)** testing of the 126-140 region (using primers 5(75)-1-F / SLA-PCR-UniRev for PCR); **(c)** testing of the 1698-1712 region (using primers 3(110)-1434-F / SLA-PCR-UniRev for PCR). The names of the primers used for reverse transcription stage (without reverse transcriptase) are labeled above the lanes. The DNA ladder is included for reference. '5(135)-Ctrl': This is the control for 5′-coterminal 18S rRNA fragments, which uses wheat RNA with the primer Ctrl-166-R for reverse transcription, and primers 5(75)-1-F and Ctrl-166-R for PCR. '3(100)-Ctrl': This is the control for 3′-terminal 18S rRNA fragments, utilizing wheat RNA, the primer Ctrl-1749-R for reverse transcription, and primers 3(110)-1434-F and Ctrl-1749-R for PCR. The primer sequences can be found in Supplementary Material S1.

S.2.2 Reactions with treatment of RNA samples with RNAse A. For the RNase-treated negative controls, total RNA (100 μg) was incubated with 10 μg of RNase A (Sigma-Aldrich, St. Louis, MO, USA) (10 mg/mL stock) at 37 °C for 30 min prior to reverse transcription."

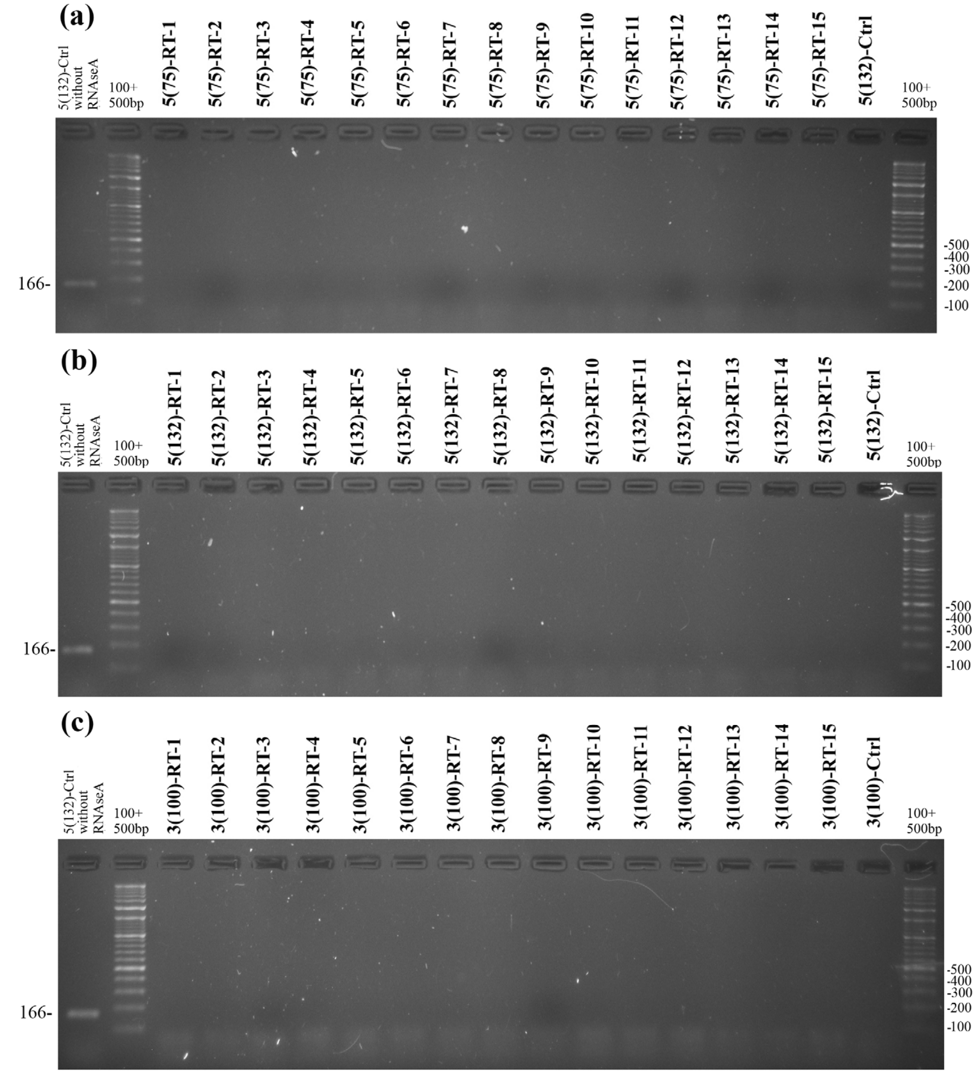

Figure S.4. Electrophoretic analysis of SLA-RT-PCR amplification products (RNA samples were treated with RNAse A prior to reverse transcription). The 1.5% agarose gels are shown (each panel represents a specific target region of the 18S rRNA). **(a)** Testing of the 75–89 region (using primers 5(75)-1-F / SLA-PCR-UniRev for PCR); **(b)** testing of the 126-140 region (using primers 5(75)-1-F / SLA-PCR-UniRev for PCR); **(c)** testing of the 1698-1712 region (using primers 3(110)-1434-F / SLA-PCR-UniRev for PCR). The names of the primers used for reverse transcription stage are labeled above the lanes. The DNA ladder is included for reference. '5(135)-Ctrl': This is the control for 5′-coterminal 18S rRNA fragments, which uses wheat RNA with the primer Ctrl-166-R for reverse transcription, and primers 5(75)-1-F and Ctrl-166-R for PCR. '3(100)-Ctrl': This is the control for 3′-terminal 18S rRNA fragments, utilizing wheat RNA, the primer Ctrl-1749-R for reverse transcription, and primers 3(110)-1434-F and Ctrl-1749-R for PCR. The primer sequences can be found in Supplementary Material S1.

S.2.3 Reactions without primers at PCR step

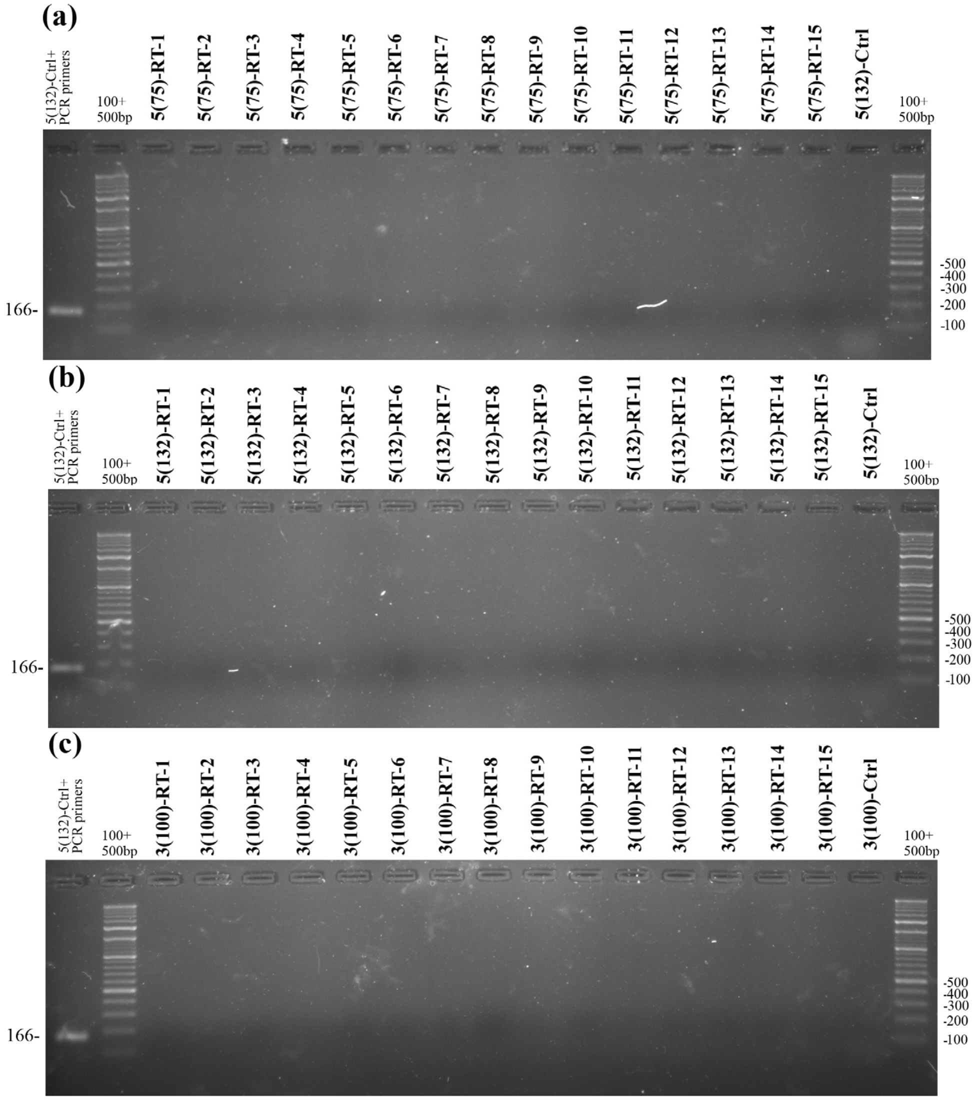

Figure S.5. Electrophoretic analysis of SLA-RT-PCR amplification products (without primers at PCR step). The 1.5% agarose gels are shown (each panel represents a specific target region of the 18S rRNA). **(a)** Testing of the 75–89 region; **(b)** testing of the 126-140 region; **(c)** testing of the 1698-1712 region. The names of the primers used for reverse transcription stage are labeled above the lanes. The DNA ladder is included for reference. ‘5(135)-Ctrl’: This is the control for 5′-coterminal 18S rRNA fragments, which uses wheat RNA with the primer Ctrl-166-R for reverse transcription. ‘3(100)-Ctrl’: This is the control for 3′-terminal 18S rRNA fragments, utilizing wheat RNA and the primer Ctrl-1749-R for reverse transcription. The primer sequences can be found in Supplementary Material S1.
